# A Causality-Informed Correlation-Aware Health-State Assessment for Complex Equipment

**DOI:** 10.3390/e28050533

**Published:** 2026-05-07

**Authors:** Wenbo Li, Zhichao Feng, Yijie Sun, Xinyi Zhang

**Affiliations:** Graduate School of Rocket Force University of Engineering, Xi’an 710025, China; liwenbo007@126.com (W.L.); 2202101097@stu.ccut.edu.cn (Y.S.); leixy@petrochina.com.cn (X.Z.)

**Keywords:** evidential reasoning rule, health-state assessment, causal coupling, convergent cross-mapping

## Abstract

Health-state assessment is a critical component of prognostics and health management (PHM) for complex equipment. Previous studies on assessing the health state of complex equipment have overlooked the statistical dependence arising from causal coupling relationships between subsystems, which is defined as causality-informed correlation in this study. This correlation introduces redundancy in health information, leading to assessment bias. To address these limitations, this study proposes a health-state assessment model based on the evidential reasoning rule considering causality-informed correlation (ERr-CIC). First, the causal coupling relationships in dynamics and their effects on health-assessment results are analyzed. Based on this analysis, the convergent cross-mapping (CCM) method is employed to examine causal coupling between subsystems. Subsequently, a health-assessment model based on ERr-CIC is developed. This model incorporates a discount factor to quantify the causality-informed correlation among indicators, realized using a conditionally hybrid correlation coefficient (CHCC), and a fusion order derived from signaling sequences. Furthermore, a sensitivity and robustness analysis of the model output to the CHCC is conducted to identify the key parameters governing system behavior and to assess the reliability of the model results under parameter perturbations. Finally, experiments are performed on the PAMD simulation device for validation, and the proposed model is compared with three other typical health-state assessment models. The results show that the ERr-CIC model proposed in this paper achieves relatively balanced performance in terms of stability and interpretability while maintaining competitive model accuracy.

## 1. Introduction

Complex equipment typically refers to precision equipment that has a complicated mechanical and electronic structure that allows for accurate measurement or control [1,2,3]. Complex equipment has wide applications in manufacturing, medical, aerospace, communications, military equipment, and many other fields. Therefore, it is important to predict the health state of complex equipment to ensure its long-term stable operation [4,5,6].

Currently, there are three main types of methods for complex equipment health-state assessment. (1) The first category is the model-based approach. The core idea of this method is to build a physical or mathematical model based on the failure modes and performance degradation mechanisms of the complex equipment and identify model parameters by reducing the error between actual and model outputs. Hu et al. [7] utilized the great likelihood estimation method to estimate the model parameters, and established a wind power bearing performance degradation model based on the Wiener process. Zhang et al. [8] established the state of predicted residuals between the weight optimization unscented Kalman filter (WOUKF) and the true capacity of the battery. (2) The second category relies on methodologies driven by data analysis. The core idea of the method is to acquire a large amount of complex equipment test data and then directly establish the mapping relationship between test statistics and health states. Jiang et al. [9] proposed a fusion of a discrete entropy-based multiscale sequence aggregation scheme and a long short-term memory neural network to predict the aero-engine’s health evolution state. Chen et al. [10] integrated the sparrow search intelligent algorithm to optimize the training effectiveness of the neural network, achieving an appropriate mean squared error between the predicted values and the actual values. (3) The third classification is based on fusion information methods. In most cases, a health-state assessment model can be developed for complex equipment based on quantitative data and qualitative knowledge. These methods mainly include: ① Belief Rule-Based approach (BRB), such as Zheng et al. [11], utilized both quantitative and qualitative information to complete health assessment for complex systems. ② Fuzzy neural network approach, Khashi et al. [12] based on the basic concepts of approximate nearest neighbor search (ANNs) and fuzzy regression modeling, proposed a new hybrid approach that allows for more accurate results in the presence of incomplete datasets. ③ The evidential reasoning approach. Based on the above analysis, “black-box” models built on quantitative data require qualitative knowledge to give them physical meaning for practical engineering applications. Methods based on semi-quantitative information enable the integrated use of quantitative data and qualitative knowledge, ensuring both the accuracy and interpretability of the assessment. As such, this paper develops a health-state assessment model for complex equipment based on a typical fusion information method, the evidential reasoning rule.

In 1994, Yang and Singh first proposed the evidential reasoning approach, which contains both quantitative and qualitative information, providing ideas for effectively solving multi-attribute decision-making problems [13,14,15]. On this basis, Yang et al. proposed the evidential reasoning rule (ER rule) in 2013 [16], which considers the weight of evidence and reliability, thereby enhancing the ER method’s capability to handle ambiguity, uncertainty, and incompleteness problems. Due to its excellent performance in integrating multi-source data and processing uncertain information, this paper establishes the health-state assessment model based on the ER rule.

However, the ER rule is valid only if the pieces of evidence are independent of one another; otherwise, they will not satisfy the commutative and associative laws, and the evidence fusion process cannot proceed. In the process of health-state assessment based on ER rule, health indicators need to be transformed into evidence and then fused. Due to the causality-informed correlation of subsystem-level health indicators, the problem of handling correlated evidence also arises. Currently, research on correlated evidence follows three directions. (1) Modifying the evidence fusion rule method. This method introduces a new evidence fusion rule that modifies existing evidence combination rules to eliminate the requirement for evidence independence [17,18,19]. (2) Methods based on relevant source evidence models. This method posits that two pieces of evidence are relevant because they were both updated from the same evidence source. Therefore, its core principle is to prevent duplicate counting of the same evidence source [20,21,22]. (3) Methods based on discount adjustment models. The core concept of this method involves applying a discounting approach to evidence, thereby transforming correlated evidence into independent evidence [23,24,25].

Based on the assessment of the health state of complex equipment, analyze the correlated evidence theories mentioned above ①. The method of modifying fusion rules can theoretically solve the impact of relevant evidence, but its current proposed method only addresses the fusion of correlated evidence in certain special situations and lacks universality. ② Method based on relevant evidence sources is relatively simple in theoretical understanding and computationally efficient. However, determining the relevant or approximately relevant evidence sources in engineering practice remains a challenging problem. ③ Method based on discount adjustment model, despite the issue that the correlation coefficient may not accurately reflect the actual dependence between pieces of evidence, is convenient for engineering applications and suitable for various scenarios involving evidence correlation. Therefore, the method based on the discounting and correction model represents a more feasible solution at the current stage. Accordingly, this paper adopts the discount adjustment model-based approach to determine the discount factor according to the causality-informed correlation among subsystem-level health indicators, and integrates this method into the ER rule to establish a health-state assessment model.

Currently, health-state assessment methods tend to explore the causal relationships behind the features of the data [26,27,28]. The purpose is to move from explaining complex “phenomena” from a statistical perspective to analyzing “causes” from a system dynamics perspective, thus improving the model’s learning efficiency and interpretability. For complex equipment, it is usually composed of multiple subsystems, and the subsystems work together in a coupled manner to accomplish a set task. Causal coupling is a relatively common type of coupling, where the output of one subsystem is the input of another subsystem. In this paper, the statistical correlation that results from the causal coupling relationship in the dynamics of the system is defined as a causality-informed correlation.

Below is a further explanation of causality-informed correlation from the perspective of complex equipment health-state assessment. The complex equipment health indicator system is typically structured into three levels: ① device-level indicator (primary indicator); ② subsystem-level indicators (secondary indicators); and ③ underlying indicators (tertiary indicators). Causality-informed correlation exists between subsystem-level indicators in the complex equipment indicator system when causal coupling is present in the system dynamics, i.e., the health information of one indicator is transmitted to another along the causal pathway, and in turn propagates further, ultimately leading to health information redundancy within the entire layer of subsystem-level indicators. Therefore, a key challenge is to identify the causal coupling relationships between subsystems and quantitatively analyze the strength of statistical dependence arising from such causal relationships, to reduce health information redundancy and improve the accuracy of health-state assessment.

Currently, there are three primary methods for conducting analysis of complex equipment internal causality coupling relationships: (1) the Granger causality analysis method. Cheng et al. [29] proposed a Granger causality analysis method based on generalized radial basis function (GRBF) neural networks for fault root cause diagnosis in industrial systems, enhancing the accuracy of diagnostic results. Zhang and Wu [30] proposed a graph neural network (GNN)-based bearing fault diagnosis method incorporating Granger causality, aiming to enhance the accuracy of bearing fault diagnosis under real-world operating conditions. (2) TF causal analysis method. Zhang et al. [31] proposed a delay-sensitive causal inference method to mitigate alarm overload issues in industrial control systems (ICS). Liu et al. [32] proposed a fault root cause analysis method based on Liang-Kleeman information flow, which can infer the location and cause of fault mechanisms by analyzing causal relationships between variables. (3) Convergent cross-mapping (CCM) method. Sharma et al. [33] applied CCM technology to nonlinear dynamic systems for detecting process anomalies or failures and identifying their root causes. Tian et al. [34] employed CCM to construct causal networks for root cause tracing in alarm systems of nonlinear industrial processes.

The three methods are compared as follows: First, the Granger causality analysis method is only applicable to linear systems. However, the system dynamic equations of complex equipment are typically nonlinear. Before applying the Granger method, the system dynamic equations must undergo linearization processing, a process that may result in the loss of critical information. Alternatively, one could use the nonlinear Granger causality method [35]; however, this method is sensitive to noise and places a heavy computational burden on multivariate systems. Subsequently, although the TF method is applicable to nonlinear systems, it requires estimating complex probability density functions. For multivariate systems, this entails a significant computational burden and poses challenges in meeting the requirements for lightweight deployment in engineering applications. In summary, the CCM method is suitable for analyzing causal relationships within complex equipment due to its high applicability to nonlinear systems and the relatively low computational burden of state-space reconstruction. A more detailed comparison of causal inference methods will be conducted in Section 6.1 using specific data.

**Figure 1:**
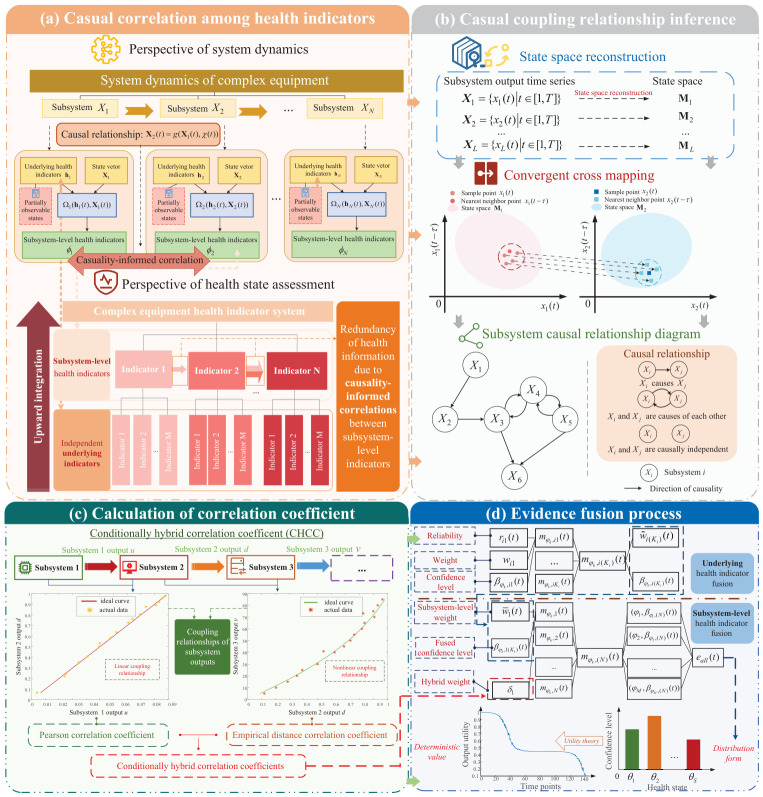
ERr-CIC modeling process.

Building upon the ER rule, this paper incorporates causality-informed correlation among subsystems to establish the evidential reasoning rule considering causality-informed correlation (ERr-CIC). The modeling process of the ERr-CIC model is illustrated in Figure 1. First, we demonstrate how causal coupling relationships between subsystems lead to causality-informed correlation among subsystem-level health indicators (Figure 1a). Second, the CCM method is employed to analyze causal coupling relationships among subsystems (Figure 1b). Subsequently, based on linear or nonlinear coupling relationships between subsystem outputs, the conditional hybrid correlation coefficient (CHCC) is calculated to quantitatively assess the magnitude of causality-informed correlation (Figure 1c). Finally, evidence fusion from the underlying health indicators to subsystem-level health indicators is performed based on the evidential reasoning rule to assess the health state of complex equipment (Figure 1d).

The innovations of this study are highlighted in several aspects. First, the introduction of causality-informed correlation effectively quantifies the causal coupling between subsystems, reducing redundancy in health information and improving assessment accuracy. Second, the CCM method is employed to identify nonlinear causal relationships among subsystems of complex equipment, providing a scientific basis for health-state assessment. Third, a health-state assessment model based on ERr-CIC is proposed, which integrates multi-indicator information through a discount factor and a CHCC, with the fusion order derived from signaling sequences to effectively handle causal correlations. Moreover, sensitivity and robustness analyses are conducted to identify key parameters and evaluate the model’s reliability under parameter perturbations. Finally, experimental validation on a PAMD simulation device, along with comparisons to typical assessment methods, demonstrates the model’s comprehensive advantages in terms of stability, interpretability, and accuracy.

## 2. Problem Formulation

This section outlines the two key problems addressed in this paper:

Problem 1: Traditional health-assessment models neglect the correlation between subsystem-level indicators. Excluding such causality-informed correlation in health-assessment methods results in redundant computation of health-state information during indicator fusion, which in turn leads to an overestimation problem that systematically degrades assessment accuracy. In this paper, the discount factor method is employed to address the causality-informed correlation among health indicators. In summary, the objective of problem 1 is to establish the following model:

(1) $$O\left(\cdot \right)=\Xi (I\left(a_{1}\right),I\left(a_{2}\right),\ldots ,I\left(a_{m}\right),\Gamma ,v)$$

where O(·) denotes the final output of the complex equipment health-state assessment model; $I\left(a_{1}\right),I\left(a_{2}\right),\ldots ,I\left(a_{m}\right)$ denote the input values of health indicators 1,2,…,m; $\Gamma =\delta_{i}|i\in \left[1,N\right]$ represents the discount factor that accounts for causality-informed correlation, with *N* being the number of subsystems; and v denotes the vector consisting of the other parameters in the health-assessment model. Problem 2: Current health-state assessment methods for complex equipment lack performance analysis specifically targeting models that account for causality-informed correlation. The objective of model performance analysis is to evaluate the impact of key parameters on model output, thereby providing data support for prognostics and health management (PHM). Analyzing the effect of causality-informed correlation among health indicators entails investigating the influence of the discount factor Γ on the health-state assessment outcome O(·) in Equation (1). This task inevitably involves complex mathematical computations and in-depth analysis of internal mechanisms, rendering it more challenging to implement.

## 3. The Modeling Process

### 3.1. Causality-Informed Correlation Among Subsystem-Level Health Indicators

This section primarily addresses how causal coupling relationships in system dynamics lead to statistical causality-informed correlation, as illustrated in Figure 1a. Below, causality-informed correlation is analyzed from the perspectives of system dynamics and health-state assessment.

(1) From the perspective of system dynamics

In the process of complex equipment health assessment, if there exists a causal relationship between subsystems, the entire system can be abstracted as a finite-dimensional coupled dynamical system:

(2) $$\dot{X}=F\left(X\right)$$

where $X\in R^{n}$ denotes the global state vector and $F\left(\cdot \right):R^{n}\to R^{n}$ is a continuously differentiable dynamical mapping. $\dot{X}=\frac{dX}{dt}$ denotes the derivative of the state vector with respect to time *t*. Assume the equipment consists of *N* subsystems $X_{1},X_{2},\ldots ,X_{N}$. The state vector can be partitioned as $X=(X_{1},X_{2},\ldots ,X_{N})$ where $X_{i}\in R^{n_{i}}$ represents the state vector of subsystem $X_{i}$, and $X_{i}=\left(x_{i1},x_{i2},\ldots ,x_{in_{i}}\right)$.

For subsystem $X_{i}$, the system dynamics can be decomposed into:

(3) $${\dot{X}}_{i}=f_{i}\left(X_{1},X_{2},\ldots ,X_{M},\chi \left(t\right)\right)$$

where $f_{i}\left(\cdot \right):R^{n}\to R^{n_{i}}$ is dynamic functions for subsystem $X_{i}$. χ(t) denotes factors other than system state, such as environmental noise and external inputs. When subsystems $X_{i}$ and $X_{j}$ exhibit causal coupling relationships, there is:

(4) $$\frac{\partial f_{i}}{\partial X_{j}}\neq 0\left(i\neq j\right)$$

This indicates that the state variables of subsystem $X_{j}$ exert a direct dynamical influence on the evolution of subsystem $X_{i}$. It can be further expressed as:

(5) $$X_{i}\left(t\right)=g\left(X_{j}\left(t\right),\chi \left(t\right)\right)$$

where g(·) denotes the state transfer function determined by system dynamics.

Based on system dynamics analysis, the following discussion addresses health-state assessment under conditions of causal relationships. Assume that the subsystem-level health indicator for subsystem $X_{i}$ is $\phi_{i}\in R$, and the underlying health indicators is $h_{i}=\left(h_{i1},h_{i2},...,h_{iK_{i}}\right)\in R^{K_{i}}$. These health indicators serve as a basis for reflecting the health state, and the subsystem health degradation evolution function is expressed as:

(6) $$\phi_{i}\left(t\right)=\Omega_{i}\left(h_{i}\left(t\right),X_{i}\left(t\right)\right)$$

where $\phi_{i}\left(t\right)$ and $h_{i}\left(t\right)$ represent the observed values of $\phi_{i}$ and $h_{i}$ at time *t*, respectively. $\Omega_{i}\left(\cdot \right):R^{K_{i}}\times R^{n_{i}}\to R$ denotes the subsystem health degradation mapping function.

**Remark 1.** 
*The underlying health indicator $h_{i}$ is essentially part of the state vector $X_{i}$. However, during health-state assessment, a subset of states from $X_{i}$ that are observable and sufficiently representative of the subsystem’s health condition are typically selected as health indicators. For clarity in subsequent reasoning, $h_{i}$ and $X_{i}$ are presented separately here.*

Similarly, for subsystem *j*, its health degradation evolution function is expressed as:

(7) $$\phi_{j}\left(t\right)=\Omega_{j}\left(h_{j}\left(t\right),X_{j}\left(t\right)\right)$$

where $\phi_{j}\left(t\right)\in R$, $h_{j}\left(t\right)\in R^{K_{j}}$, and $X_{j}\left(t\right)\in R^{n_{j}}$ represent the subsystem-level health indicators, underlying health indicators, and state vector for subsystem $X_{j}$, respectively. Since $X_{i}$ depends on $X_{j}$, and $\phi_{i}\left(t\right)=\Omega_{i}\left(h_{i}\left(t\right),X_{i}\left(t\right)\right)$, therefore $\phi_{i}$ will be affected by $X_{j}$.

(2) From the perspective of health assessment

As shown in the preceding analysis, subsystem-level indicator $\phi_{i}$ is influenced by $\phi_{j}$. This relationship leads to redundancy in health information during the health-state assessment process, as explained below. Health state Ω can be understood as a latent variable reflecting the degradation of the equipment’s health. During the health-state assessment process, the amount of information that $\phi_{i}$ and $\phi_{j}$ can provide to Ω is

(8) $$I\left(\Omega ;\phi_{i},\phi_{j}\right)=H\left(\Omega \right)-H\left(\Omega \left|\phi_{i},\phi_{j}\right.\right)$$

where H(Ω) represents the entropy of Ω. $H(\Omega \left|\phi_{i},\phi_{j}\right.)$ denotes the entropy of Ω given the observation of $\phi_{i}$ and $\phi_{j}$. $I(\Omega ;\phi_{i},\phi_{j})$ is the mutual information between Ω, $\phi_{i}$ and $\phi_{j}$, indicating the extent to which the uncertainty regarding Ω is reduced after observing $\phi_{i}$ and $\phi_{j}$.

If $\phi_{i}$ and $\phi_{j}$ are independent of each other, then $I(\Omega ;\phi_{i},\phi_{j})$ can be approximated as:

(9) $$I\left(\Omega ;\phi_{i},\phi_{j}\right)=I\left(\Omega ;\phi_{i}\right)+I\left(\Omega ;\phi_{j}\right)$$

However, since there is redundancy in health information between $\phi_{i}$ and $\phi_{j}$, therefore $I\left(\Omega ;\phi_{i},\phi_{j}\right)<I\left(\Omega ;\phi_{i}\right)+I\left(\Omega ;\phi_{j}\right)$. Redundant health information is represented as

(10) $$R\left(\phi_{i},\phi_{j}\right)=I\left(\Omega ;\phi_{i}\right)+I\left(\Omega ;\phi_{j}\right)-I\left(\Omega ;\phi_{i},\phi_{j}\right)$$

where $R(\phi_{i},\phi_{j})$ represents redundant health information between $\phi_{i}$ and $\phi_{j}$. Since $I\left(\Omega ;\phi_{i},\phi_{j}\right)=I\left(\Omega ;\phi_{i}\right)+I\left(\Omega ;\phi_{i}\left|\phi_{j}\right.\right)$, Equation (10) can be rewritten as

(11) $$R\left(\phi_{i},\phi_{j}\right)=I\left(\Omega ;\phi_{j}\right)-I\left(\Omega ;\phi_{i}\left|\phi_{j}\right.\right)$$

It is evident that $R(\phi_{i},\phi_{j})$ essentially depends on the degree of information dependence between $\phi_{i}$ and $\phi_{j}$, i.e., the magnitude of the mutual information $I(\phi_{i},\phi_{j})$ and $I(\phi_{i},\phi_{j})$ is directionless. At the same time, $I(\phi_{i},\phi_{j})$ satisfies the mapping relationship described below.

(12) $$I\left(\phi_{i},\phi_{j}\right)=f\left(K\left(\phi_{i},\phi_{j}\right)\right),\frac{df}{dK}>0$$

where $K(\phi_{i},\phi_{j})$ denotes the correlation coefficient of $\phi_{i}$ and $\phi_{j}$. f(·) indicates the mapping function from $K(\phi_{i},\phi_{j})$ to $I(\phi_{i},\phi_{j})$.

Therefore, redundant health information can be reflected by the correlation coefficient. Based on the above analysis, the causal relationship between $\phi_{i}$ and $\phi_{j}$ leads to redundancy in health information during the health-state assessment process, and this redundant health information can be reflected by the correlation coefficient. This correlation is referred to as causality-informed correlation in this paper.

Based on the above analysis, calculating causality-informed correlation between subsystems requires addressing the following two problems: ① Determining whether dynamic causal coupling relationships exist between subsystems; ② Quantifying the magnitude of causality-informed correlation among health indicators.

### 3.2. Convergent Cross-Mapping for Causal Relationships Inference

Sugihara proposed the convergent cross-mapping (CCM) method in 2012. For complex equipment, the intricate coupling relationships among internal subsystems often make it difficult to establish accurate parametric models. CCM can detect directed causal relationships within coupled dynamic systems without requiring explicit parametric models, making it well-suited for identifying causal relationships between subsystems in complex equipment.

Based on the findings of Butler [36] and Cummins [37] on the applicability of state-space reconstruction (SSR) and convergent cross-mapping (CCM), the conditions for subsystems to be eligible for causal coupling analysis are established as follows: Condition ①: Each subsystem performs a single function, meaning a subsystem ultimately has only one output. The main purpose of dividing subsystems according to a single output is to ensure the clarity of the causal analysis chain, thereby making it easier to identify the dominant factor at any given moment. Condition ②: No closed-loop feedback structures exist between subsystems. According to Butler’s research, if there is a feedback loop between two objects, their influences on each other will be coupled, making it impossible to distinguish which one is the driving factor, thereby rendering causal analysis meaningless. Condition ③: External inputs to a subsystem can be treated as constants, i.e., $\chi \left(t\right)=\chi_{0}$, where $\chi_{0}$ is a constant parameter. If the system is subject to external inputs that change over time, it is impossible to form a stable manifold, and consequently, state-space reconstruction cannot be performed, rendering the basic conditions for the CCM method invalid.

The process of determining the causal coupling relationship within the subsystem is illustrated in Figure 1b. Assume that the time series generated by projecting systems $X_{i}$ and $X_{j}$ onto a one-dimensional space is: $Y_{i}=\left\{Y_{i}\left(t\right)\left|t\right.\in \left[1,T\right]\right\}$, $Y_{j}=\left\{Y_{j}\left(t\right)\left|t\right.\in \left[1,T\right]\right\}$. According to the Takens embedding theorem [38], let the embedding dimension be *m* and the delay time be τ. The reconstructed state vector is represented as:

(13) $${\tilde{Y}}_{i}\left(t\right)=\left[Y_{i}\left(t\right),Y_{i}\left(t-\tau \right),\ldots ,Y_{i}\left(t-\left(m-1\right)\tau \right)\right]$$

(14) $${\tilde{Y}}_{j}\left(t\right)=\left[Y_{j}\left(t\right),Y_{j}\left(t-\tau \right),\ldots ,Y_{j}\left(t-\left(m-1\right)\tau \right)\right]$$

where *m* and τ are fixed positive integers. *m* is determined using the pseudo-neighborhood method [39], and τ is determined using the average mutual information method [40]. The reconstructed manifolds are, respectively: $M_{i}=\left\{{\tilde{Y}}_{i}\left(t\right)\left|t=(m-1)\tau +1,\ldots ,T\right.\right\}$, $M_{i}=\left\{{\tilde{Y}}_{j}\left(t\right)\left|t=(m-1)\tau +1,\ldots ,T\right.\right\}$.

According to research by Butler et al., when the reconstructed state space satisfies both Auto-predictability fraction (AF) and Recurrence fraction (RF) metrics on top of meeting the conditions mentioned earlier, CCM can be employed to determine causal relationships. Specifically, AF and RF should approach 1. For detailed calculation methods, please refer to reference [36], and further elaboration is omitted here.

For a specific time *t*, find the Q=m+1 nearest neighbor points ${\left\{{\tilde{Y}}_{i}\left(t_{q}\right)\right\}}_{q=1}^{Q}$ on $M_{i}$ that are closest to ${\tilde{Y}}_{i}\left(t\right)$, with the corresponding time index being $t_{k}$. Map ${\left\{{\tilde{Y}}_{i}\left(t_{q}\right)\right\}}_{q=1}^{Q}$ to $Y_{j}=\left\{Y_{j}\left(t\right)\left|t\right.\in \left[1,T\right]\right\}$, where the corresponding sample point is ${\left\{Y_{j}\left(t_{k}\right)\right\}}_{k=1}^{K}$. Calculate the estimated value ${\hat{Y}}_{j}\left(t\right)$ of $Y_{j}\left(t\right)$.

(15) $${\hat{Y}}_{j}\left(t\right)=\sum_{q=1}^{Q}v_{q}Y_{j}\left(t_{q}\right)$$

(16) $$v_{q}=\frac{\exp(-d_{q}/d_{1})}{\sum_{\iota =1}^{Q}\exp(-d_{\iota }/d_{1})}$$

(17) $$d_{q}=∥\tilde{Y}\left(t\right)-\tilde{Y}\left(t_{q}\right)∥$$

where $∥\tilde{Y}\left(t\right)-\tilde{Y}\left(t_{q}\right)∥$ denotes Euclidean distance. $d_{1}$ represents the nearest neighbor distance. Define ${\hat{Y}}_{j}\left(t\right)$ as the cross-mapping from $Y_{i}$ to $Y_{j}$ of $Y_{j}\left(t\right)$. Calculate the correlation coefficient $r_{ij}$ between ${\hat{Y}}_{j}\left(t\right)$ and $Y_{j}\left(t\right)$ using the equation:

(18) $$r_{ij}=\frac{\sum_{t=1}^{Z}\left(Y_{j}\left(t\right)-{\bar{Y}}_{j}\left(t\right)\right)\left({\hat{Y}}_{j}\left(t\right)-{\bar{Y}}_{j}^{\prime }\left(t\right)\right)}{\sqrt{\sum_{t=1}^{Z}{\left(Y_{j}\left(t\right)-{\bar{Y}}_{j}\left(t\right)\right)}^{2}\sum_{t=1}^{Z}{\left({\hat{Y}}_{j}\left(t\right)-{\bar{Y}}_{j}^{\prime }\left(t\right)\right)}^{2}}}$$

where *Z* denotes the sample length. ${\bar{Y}}_{j}\left(t\right)$, ${\bar{Y}}_{j}^{\prime }\left(t\right)$ represent the mean of the actual values and the mean of the estimated values, respectively. As the sample length *Z* increases, ${\hat{Y}}_{j}\left(t\right)$ gradually converges toward $Y_{j}\left(t\right)$, and the correlation coefficient ultimately converges to [0, 1]. This convergence suggests the existence of a causal relationship from subsystem $X_{i}$ to subsystem $X_{j}$, represented as $X_{i}\to X_{j}$; The coefficient $r_{ji}$ is then computed to assess the reverse causality. Specifically, if $r_{ji}\in \left[0,1\right]$, there is $X_{j}\to X_{i}$. Conversely, if $r_{ij},r_{ji}\notin \left[0,1\right]$, no causal relationship is inferred between $X_{i}$ and $X_{j}$, denoted as $X_{i}⊥X_{j}$. This paper represents the causal relationships between subsystems in the form of a directed acyclic graph (DAG), referred to as a causal relationship graph.

**Definition 1.** 
*Subsystem causal relationship graph G=(V,E), $V=\{V_{i}\left|i\in [1,M]\right.\}$ is the set of vertices. $V_{i}$ corresponds to subsystem $X_{i}$; $E=\{E_{ij}\left|i,j\in [1,M]\right.\}$ is the set of directed edges connecting the vertices, used to indicate the causal direction between two subsystems.*

**Figure 2:**
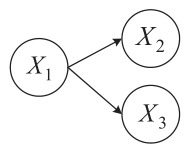
Causal relationship graph example.

For example, suppose the causal relationships among subsystems $X_{1}$, $X_{2}$, and $X_{3}$ are as follows: $X_{1}\to X_{2}$, $X_{1}\to X_{3}$, $X_{2}⊥X_{3}$. Then the causal relationship graph of the subsystems is represented as Figure 2.

### 3.3. Calculation of Conditionally Hybrid Correlation Coefficients

In Section 3.2, the analysis of causal relationships among subsystems is conducted using the CCM method. However, CCM can only determine the direction of causality and cannot quantitatively analyze the magnitude of the causal driving effect between subsystems. This poses a challenge for all current causal analysis methods. However, for the subsequent health-state assessment task, what is needed is not the magnitude of the causal effect itself, but the strength of statistical dependence among subsystem-level indicators induced by the identified coupling structure. This dependence encompasses both linear and nonlinear correlations. This paper utilizes CHCC to represent the correlation between indicators. The calculation process is shown in Figure 1c.

Assume the causal relationship between subsystems $X_{i}$ and $X_{j}$ is $X_{i}\to X_{j}$. The one-dimensional projection of state space of subsystems $X_{i}$ and $X_{j}$ are $Y_{i}$ and $Y_{j}$. From the perspective of system dynamics, if the relationship between $Y_{i}$ and $Y_{j}$ is linear, which means $Y_{i}$ and $Y_{j}$ satisfy the relation equation:

(19) $$Y_{j}=aY_{i}+b$$

where *a*, *b* are arbitrary constants. Then Pearson’s correlation coefficient is used to calculate the correlation coefficient between $Y_{i}$ and $Y_{j}$.

(20) $$L\left(Y_{i},Y_{j}\right)=\frac{\sum_{t=1}^{N}\left(Y_{i}\left(t\right)-{\bar{Y}}_{i}\right)\left(Y_{j}\left(t\right)-{\bar{Y}}_{j}\right)}{\sigma_{i}\sigma_{j}}$$

where $L(Y_{i},Y_{j})$ denotes linear correlation coefficient. ${\bar{Y}}_{i}$ and ${\bar{Y}}_{j}$ indicate the average of $Y_{i}$ and $Y_{j}$, respectively. $\sigma_{i}$, $\sigma_{j}$ stand for the variance of $Y_{i}$ and $Y_{j}$.

When there is a nonlinear relationship between $X_{i}$ and $X_{j}$, the correlation coefficient is calculated using the empirical distance covariance [41]:

(21) $$R\left(Y_{i},Y_{j}\right)=\frac{v^{2}\left(Y_{i},Y_{j}\right)}{\sqrt{v^{2}\left(Y_{i},Y_{i}\right)v^{2}\left(Y_{j},Y_{j}\right)}}$$

where $R(Y_{i},Y_{j})$ denotes nonlinear correlation coefficient. $v^{2}\left(Y_{i},Y_{j}\right)$, $v^{2}\left(Y_{i},Y_{i}\right)$, $v^{2}\left(Y_{j},Y_{j}\right)$ stand for the empirical distance covariance between $\left(Y_{i},Y_{j}\right)$, $\left(Y_{i},Y_{i}\right)$, and $\left(Y_{j},Y_{j}\right)$, respectively.

Specifically, the calculation of the correlation coefficient is determined by the function $F(Y_{i},Y_{j})$:

(22) $$K\left(Y_{i},Y_{j}\right)=F\left(Y_{i},Y_{j}\right)=\left\{\begin{array}{cc} L(Y_{i},Y_{j}) & Y_{j}=aY_{i}+b\\ R(Y_{i},Y_{j}) & else \end{array}\right.$$

where $K(Y_{i},Y_{j})$ is a generalized representation of the correlation coefficient between $Y_{i}$ and $Y_{j}$.

In practice, the following methods can be used to determine whether the relationship between $Y_{i}$ and $Y_{j}$ is linear or nonlinear. First, perform a linear regression fit on $Y_{j}$ to obtain the residual sequence ε.

(23) $$Y_{j}=aY_{i}+b+\varepsilon$$

(24) $$\varepsilon =Y_{j}-{\hat{Y}}_{j}$$

where ${\hat{Y}}_{j}$ represents the fitted value. ε indicates the residual sequence. If $Y_{i}$ and $Y_{j}$ are linearly related, then ε should not contain any systematic structure. Calculate the empirical distance correlation coefficient between $Y_{i}$ and ε. The criterion for determining whether the relationship between $Y_{i}$ and $Y_{j}$ is linear or nonlinear is

(25) $$\left\{\begin{array}{cc} R(Y_{i},\varepsilon )<\iota & \mathrm{linear}\\ R(Y_{i},\varepsilon )⩾\iota & \mathrm{nolinear} \end{array}\right.$$

ι represents a minimum value constant.

Without loss of generality, define the projection vector of the *N* subsystems $X_{1},X_{2},\ldots ,X_{N}$ as $Y_{1},Y_{2},\ldots ,Y_{N}$. The correlation matrix which denoted as **K** is obtained as follows:

(26) $$K=\left[\begin{array}{cccc} K(Y_{1},Y_{1}) & & & \\ K(Y_{2},Y_{1}) & K(Y_{2},Y_{2}) & & \\ \ldots & \ldots & \ldots & \\ K(Y_{N},Y_{1}) & K(Y_{N},Y_{2}) & \ldots & K(Y_{N},Y_{N}) \end{array}\right]$$

The initial correlation coefficient $k_{i}$ for subsystem $X_{i}$ is:

(27) $$k_{i}=\sum_{j=1}^{i}K(Y_{i},Y_{j})$$

Then the CHCC $\delta_{i}$ of $X_{i}$ is:

(28) $$\delta_{i}=\frac{1/k_{i}}{\sum_{i=1}^{N}1/k_{i}}$$

**Remark 2.** 
*The parameter $k_{i}$ increases monotonically with the indicator correlation strength. When calculating the discount factor for an indicator, $k_{i}$ is inverted and normalized so that the larger the value of $k_{i}$, the smaller the value of $\delta_{i}$, and accordingly, the discount for that indicator is approximately larger. $\delta_{i}$ takes values in the range of [0, 1]. $\delta_{i}$ = 0 means that the health information of subsystem $X_{i}$ can be completely represented by other subsystems. $\delta_{i}$ = 1 means that subsystem $\delta_{i}$ is completely independent from other subsystems. $0<\delta_{i}<1$ means that subsystem $X_{i}$ has causality-informed correlation with other subsystems.*

### 3.4. Calculation of Other Parameters

For the subsystem $X_{i}$ with causality-informed correlation, it consists of $K_{i}$ mutually independent underlying indicators $h_{i}\in R^{K_{i}}$. In the ER rule, the process of transforming indicators into evidence requires the determination of three other key parameters: confidence level, weight, and reliability.

- Calculation of confidence level β

Assume there are M health grade, represented as: $\Theta =\{\varphi_{k}\left|k\in [1,M]\right.\}$. Θ is called the assessment framework.

(29) $$\beta_{\varphi_{k},iz}\left(t\right)=\left\{\begin{array}{cc} \frac{U\left(\varphi_{n+1}\right)-h_{iz}\left(t\right)}{U\left(\varphi_{n+1}\right)-U\left(\varphi_{n}\right)} & k=n,\;\mathrm{if}\;U\left(\varphi_{n}\right)⩽h_{iz}\left(t\right)⩽U\left(\varphi_{n+1}\right)\\ \frac{h_{iz}\left(t\right)-U\left(\varphi_{n}\right)}{U\left(\varphi_{n+1}\right)-U\left(\varphi_{n}\right)} & k=n+1\\ 0 & k\neq n,n+1 \end{array}\right.$$

where $\beta_{\varphi_{k},iz}\left(t\right)$ represents the probability that indicator $h_{iz}\left(i\in \left[1,N\right],z\in \left[1,K_{i}\right]\right)$ is assessed as health grade $\varphi_{k}$ at time *t*, which is called confidence level in the ER rule. $U(\varphi_{k})$ represents the reference value for health grade $\varphi_{k}$ and meets $U\left(\varphi_{1}\right)<U\left(\varphi_{2}\right)<,\ldots ,<U\left(\varphi_{M}\right)$. $h_{iz}\left(t\right)$ represents the monitored value of indicator $h_{iz}$ at time *t*. Through the above processing, health indicator $h_{iz}$ monitoring data can be transformed into an evidence-distribution form.

(30) $$e_{iz}\left(t\right)=\left\{\left(\varphi_{k},\beta_{\varphi_{k},iz}\left(t\right)\right)\left|\forall \varphi_{k}\subseteq \Theta \right.\right\}$$

where $e_{iz}\left(t\right)$ represents the evidence distribution of indicator $h_{iz}$ at time *t*.

- Calculation of weight *w*

The coefficient of the variation-based weighting (CVBW) can effectively capture the fluctuation of indicators [42], which reflects the level of attention given to each indicator. Therefore, this study employs CVBW to calculate the weights of the underlying indicators.

Indicator weight $w_{iz}$ is calculated as:

(31) $$w_{iz}=\varepsilon_{iz}/\sum_{\iota =1}^{K_{i}}\varepsilon_{i\iota }$$

(32) $$\varepsilon_{iz}=\sigma_{iz}/{\bar{h}}_{iz}$$

where ${\bar{h}}_{iz}$, $\sigma_{i}z$ denote the mean and standard deviation of the monitoring data of $h_{iz}$, respectively. $\varepsilon_{iz}$ represents the coefficient of variation.

- Calculation of the reliability *r*

The reliability of the evidence $r_{iz}\left(t\right)$ consists of a static reliability ${r_{iz}}^{S}$ and a dynamic reliability ${r_{iz}}^{D}\left(t\right)$. The exact value of the static reliability ${r_{iz}}^{S}$ is given by the expert. Dynamic reliability ${r_{iz}}^{D}\left(t\right)$ is calculated using the distance-based calculation method [43]. As indicated in reference [44], the evidence reliability *r* can be derived by combining the static reliability ${r_{iz}}^{S}$ and dynamic reliability ${r_{iz}}^{D}\left(t\right)$ through the perturbation coefficient. The specific calculation method is beyond the focus of this paper and will not be discussed further.

### 3.5. Evidence Fusion Process

This section obtains the health-state assessment results of complex equipment through evidence fusion from underlying indicators to subsystem-level indicators, as shown in Figure 1d.

According to the ER rule [16], before performing evidence fusion, the evidence needs to be converted into the weighted belief distribution with reliability (WBDR) form. The elements of WBDR are called basic probability mass, and the basic probability mass of evidence $e_{iz}\left(t\right)$ is represented as

(33) $$m_{\varphi_{k},iz}\left(t\right)=\left\{\begin{array}{cc} 0 & \varphi_{k}=\emptyset \\ {\tilde{w}}_{iz}\left(t\right)\beta_{\varphi_{k},iz}\left(t\right) & \varphi_{k}\subseteq \Theta ,\varphi_{k}\neq \emptyset \\ 1-{\tilde{w}}_{iz}\left(t\right) & \varphi_{k}=P\left(\Theta \right) \end{array}\right.$$

(34) $${\tilde{w}}_{iz}\left(t\right)=\frac{w_{iz}}{1+w_{iz}+r_{iz}\left(t\right)}$$

where P(Θ) denotes the power set of Θ. ${\tilde{w}}_{iz}\left(t\right)$ indicates the hybrid weight of health indicator $h_{iz}$.

The WBDR can be represented by

(35) $$m_{iz}\left(t\right)=\left\{\left(\varphi_{k},m_{\varphi_{k},iz}\left(t\right)\right),\forall \varphi_{k}\subseteq \Theta ;\left(P\left(\Theta \right),m_{P(\Theta ),iz}\left(t\right)\right)\right\}$$

The evidence is integrated in a pairwise fusion manner, and the specific process of the final fusion is shown as Equations (31)–(33).

(36) $${\hat{m}}_{\varphi_{k},i\left(K_{i}\right)}\left(t\right)=\left[\left(1-r_{K_{i}}\left(t\right)\right){\hat{m}}_{\varphi_{k},i\left(K_{i}-1\right)}\left(t\right)+{\hat{m}}_{P\left(\Theta \right),i\left(K_{i}-1\right)}\left(t\right)m_{\varphi_{k},iK_{i}}\left(t\right)\right]$$

(37) $${\hat{m}}_{P\left(\Theta \right),i\left(K_{i}\right)}\left(t\right)=\left(1-r_{iK_{i}}\left(t\right)\right){\hat{m}}_{P\left(\Theta \right),i\left(K_{i}-1\right)}\left(t\right)$$

(38) $$m_{\varphi_{k},i\left(K_{i}\right)}\left(t\right)=\frac{{\hat{m}}_{\varphi_{k},i\left(K_{i}\right)}\left(t\right)}{\sum_{E\subseteq \Theta }{\hat{m}}_{E,i(K_{i})}\left(t\right)+{\hat{m}}_{P\left(\Theta \right),i\left(K_{i}\right)}}$$

where ${\hat{m}}_{\varphi_{k},i\left(K_{i}\right)}\left(t\right)$ denote the unnormalized basic probability mass after fusing the $K_{i}$ health indicators of subsystem $X_{i}$ and assessed as health grade $\varphi_{k}$. ${\hat{m}}_{P\left(\Theta \right),i\left(K_{i}\right)}$ represents the unnormalized basic probability mass of the power set. Similarly, ${\hat{m}}_{\varphi_{k},i\left(K_{i}-1\right)}\left(t\right)$ and ${\hat{m}}_{P\left(\Theta \right),i\left(K_{i}-1\right)}\left(t\right)$ indicate the fusion results of $K_{i}-1$ indicators. $m_{\varphi_{k},i\left(K_{i}\right)}\left(t\right)$ is the normalized basic probability mass.

The confidence level for health grade $\varphi_{k}$ after fusing $K_{i}$ underlying indicators is

(39) $$\beta_{\varphi_{k},i\left(K_{i}\right)}\left(t\right)=\frac{{\hat{m}}_{\varphi_{k},i\left(K_{i}\right)}\left(t\right)}{\sum_{E\subseteq \Theta }{\hat{m}}_{E,i(K_{i})}\left(t\right)}$$

According to Equation (28), the hybrid weight after the fusion of $K_{i}$ indicators is calculated as

(40) $${\tilde{w}}_{i(K_{i})}\left(t\right)=\frac{m_{\varphi_{k},i\left(K_{i}\right)}\left(t\right)}{\beta_{\varphi_{k},i\left(K_{i}\right)}\left(t\right)}=\frac{\sum_{E\subseteq \Theta }{\hat{m}}_{E,i(K_{i})}\left(t\right)}{\sum_{E\subseteq \Theta }{\hat{m}}_{E,i(K_{i})}\left(t\right)+{\hat{m}}_{P\left(\Theta \right),i\left(K_{i}\right)}\left(t\right)}$$

where ${\tilde{w}}_{i(K_{i})}\left(t\right)$ denote the hybrid weight after the fusion of $K_{i}$ indicators.

According to Equation (35), the fusion results of the hybrid weights of the underlying indicators of each subsystem ${\tilde{w}}_{1(K_{1})}\left(t\right),{\tilde{w}}_{2(K_{2})}\left(t\right),\ldots ,{\tilde{w}}_{N(K_{N})}\left(t\right)$ can be obtained. Normalize the above hybrid weights to obtain subsystem-level health indicator weights.

(41) $${\bar{w}}_{i}\left(t\right)={\tilde{w}}_{i(K_{i})}\left(t\right)/\sum_{\iota =1}^{N}{\tilde{w}}_{\iota (K_{\iota })}\left(t\right)$$

where ${\bar{w}}_{i}\left(t\right)$ indicates the weight of subsystem-level health indicator $\phi_{i}$.

**Remark 3.** 
*If the hybrid weights obtained from the fusion of underlying indicators are directly used as the weights for subsystem-level health indicators, it is essentially equivalent to directly fusing the underlying indicators of all subsystems. Reference [45] demonstrates that when using the ER rule for evidence fusion, the more evidence is fused, the greater the risk of overfitting the fusion result. Therefore, this paper adopts normalization to reduce the risk of overfitting.*

In summary, the WBDR of subsystem-level health indicator $\phi_{i}$ can be expressed as:

(42) $$m_{i}\left(t\right)=\left\{\left(\varphi_{k},m_{\varphi_{k},i}\left(t\right)\right),\forall \varphi_{k}\subseteq \Theta ;\left(P\left(\Theta \right),m_{P(\Theta ),i}\left(t\right)\right)\right\}$$

(43) $$m_{\varphi_{k},i}\left(t\right)=\left\{\begin{array}{cc} 0 & \varphi_{k}=\emptyset \\ {\bar{w}}_{i}\left(t\right)\delta_{i}\beta_{\varphi_{k},i\left(K_{i}\right)}\left(t\right) & \varphi_{k}\subseteq \Theta ,\varphi_{k}\neq \emptyset \\ 1-{\bar{w}}_{i}\left(t\right) & \varphi_{k}=P\left(\Theta \right) \end{array}\right.$$

where $m_{i}\left(t\right)$ denote the WBDR of subsystem-level health indicator $\phi_{i}$. $m_{\varphi_{k},i}\left(t\right)$ represents the basic probability mass of $\phi_{i}$ being assessed at health grade $\varphi_{k}$. $\delta_{i}$ indicates the CHCC of $\phi_{i}$.

By repeating the fusion process of Equations (31)–(33), performing evidence fusion on subsystem-level health indicators. The fusion process is abbreviated as follows:

(44) $$m_{\varphi_{k},\left(N\right)}\left(t\right)=m_{\varphi_{k},1}\left(t\right)⊗m_{\varphi_{k},2}\left(t\right)⊗,\ldots ,m_{\varphi_{k},N}\left(t\right)$$

where $m_{\varphi_{k},\left(N\right)}\left(t\right)$ denotes the basic probability mass assigned to the health grade $\varphi_{k}$ after the fusion of *N* subsystem-level health indicators. ⊗ represents the fusion symbol.

Based on Equation (34), the confidence level for health grade $\varphi_{k}$ after fusing *N* subsystem-level indicators is

(45) $$\beta_{\varphi_{k},\left(N\right)}=\frac{{\hat{m}}_{\varphi_{k},\left(N\right)}\left(t\right)}{\sum_{E\subseteq \Theta }{\hat{m}}_{E,(N)}\left(t\right)}$$

Ultimately, it can yield the evidence distribution (or referred to as the health-state distribution) results of complex equipment.

(46) $$e_{all}\left(t\right)=\left\{\left(\varphi_{k},\beta_{\varphi_{k},\left(N\right)}\left(t\right)\right)\left|\forall \varphi_{k}\subseteq \Theta \right.\right\}$$

where $e_{all}\left(t\right)$ denotes the evidence distribution of complex equipment. $\beta_{\varphi_{k},\left(N\right)}\left(t\right)$ represents the confidence level that complex equipment is assessed as health grade $\varphi_{k}$. (N) represents the fusion of *N* subsystem-level health indicators.

Since the evidence is correlated, the evidence fusion will no longer satisfy the law of exchange and the law of union. The problem of determining the fusion order is needed when evidence with correlation is fused [46]. This paper proposes a method to determine the fusion order based on the signaling sequence.

Let the signaling relationship between the subsystems $X_{1},X_{2},\ldots ,X_{N}$ with causality-informed correlation be: the output of $X_{1}$ is the input of $X_{2}$ and the output of $X_{2}$ serve as the input of $X_{3}$…Then, the signaling sequence is: 1,2,…,N. Seq(i) denotes the signaling sequence number of subsystem $X_{i}$.

Let SeqF(i) denote the fusion order of $S_{i}$, and SeqF(i)=1 represents that piece of evidence is fused first. Then there are:

(47) SeqF(i)=Seq(i)

The fusion order SeqF(i) reflects the priority assigned to each indicator, where a higher priority indicates greater attention. For indicators with causality-informed correlation, the health information of upstream indicators in the signaling sequence is transmitted downstream. Accordingly, upstream indicators should be assigned higher priority. The fusion order follows the signaling sequence derived from the causal relationship graph and equipment working mechanism, corresponding to a topological ordering that ensures causally consistent information propagation and gives sufficient priority to upstream indicators.

The distribution form shown in Equation (41) is not conducive to model optimization or comparison. Therefore, further defuzzification processing is required to convert the distribution form into a deterministic numerical form. According to the utility theory proposed by Yang [14], the output utility of complex equipment is:

(48) $$\eta \left(t\right)=\sum_{\varphi_{k}\subseteq \Theta }\eta \left(\varphi_{k}\right)\beta_{\varphi_{k},\left(N\right)}\left(t\right)$$

where $\eta (\varphi_{k})$ is the utility value of health grade $\varphi_{k}$ and is typically obtained based on expert knowledge or statistical results from actual engineering data. η(t) indicates the output utility of the complex equipment at time *t*.

### 3.6. Optimization of Model Parameters

The parameters of ERr-CIC $\Gamma =\{\delta_{i}\left|i\in [1,N]\right.\}$, $U=\{U\left(\varphi_{k}\right)\left|k\in [1,M]\right.\}$, and $\Psi =\{\eta \left(\varphi_{k}\right)\left|k\in [1,M]\right.\}$ are calculated based on the indicator monitoring data or determined by expert knowledge. However, the adaptability of the model parameters decreases due to factors such as disturbances and environmental changes that are inevitable during complex equipment health monitoring. Therefore, the model needs to be trained with multiple sets of data to optimize the model parameters.

Mean squared error (MSE) is widely used for evaluating model accuracy. It quantifies the precision of the ERr-CIC model by calculating the average of the squares of the difference between the actual output utility η(t) and the expected output utility $\eta_{\exp}\left(t\right)$. $\eta_{\exp}\left(t\right)$ is typically determined by domain experts based on the evaluation scenario, historical statistical analysis, and industry standards. The optimization objective functions are defined as:

(49) $$\min_{\Gamma ,U,\Psi }MSE\left(\eta \left(t\right)-\eta_{\exp}\left(t\right)\right)$$

The corresponding parameter constraints are shown in Equations (45)–(47).

(50) $$0⩽\delta_{i}⩽1,\sum_{i=1}^{N}\delta_{i}=1$$

(51) $$U\left(\varphi_{1}\right)<U\left(\varphi_{2}\right)<,\ldots ,U\left(\varphi_{k}\right)$$

(52) $$\eta \left(\varphi_{1}\right)<\eta \left(\varphi_{2}\right)<,\ldots ,\eta \left(\varphi_{k}\right)$$

## 4. Sensitivity and Robustness Analysis

To improve the model’s interpretability and provide a quantitative analysis of model parameters for practical engineering applications, this section presents a sensitivity and robustness analysis.

### 4.1. Sensitivity Analysis

For problem 2, based on the reasoning process outlined earlier, this section analyzes the sensitivity of the output confidence level to CHCC within the complex equipment. In addition, through the mathematical derivation process of the sensitivity analysis, the traceability analysis of the model output is also realized.

**Proposition 1.** 
*According to engineering practice, the less dependent an indicator is on other indicators, the greater its impact on the overall health state. In the ERr-CIC model, a higher CHCC of an indicator at the same evaluation level corresponds to a higher overall output utility of the equipment. Thus, it is speculated that a negative correlation exists between indicator causality-informed correlation and complex equipment health state.*

**Proof of the Proposition 1.**  The sensitivity analysis is aimed at the sensitivity of the output to the relative overall correlation coefficient (in the case of $\delta_{i}$), denoted as $\frac{\partial \beta_{\varphi_{k},\left(N\right)}\left(t\right)}{\partial \delta_{i}}$. For convenience of notation, the variable *t* will be omitted in the subsequent derivation:

(53) $$\frac{\partial \beta_{\varphi_{k},\left(N\right)}}{\partial \delta_{i}}=\left[\frac{\partial \beta_{\varphi_{k},\left(N\right)}}{\partial {\hat{m}}_{\varphi_{k},\left(N\right)}},\frac{\partial \beta_{\varphi_{k},\left(N\right)}}{\partial {\hat{m}}_{P(\Theta ),(N)}}\right]\cdot \left[\begin{array}{c} \frac{\partial {\hat{m}}_{\varphi_{k},\left(N\right)}}{\partial \delta_{i}}\\ \frac{\partial {\hat{m}}_{P(\Theta ),(N)}}{\partial \delta_{i}} \end{array}\right]$$

(54) $$\begin{array}{c} \frac{\partial {\hat{m}}_{\varphi_{k},\left(N\right)}}{\partial \delta_{i}}=\\ \left[\begin{array}{c} \frac{\partial {\hat{m}}_{\varphi_{k},\left(N\right)}}{\partial m_{P(\Theta ),i}}\\ \frac{\partial {\hat{m}}_{\varphi_{k},\left(N\right)}}{\partial {\hat{m}}_{\varphi_{k},\left(N-1\right)}}\\ \frac{\partial {\hat{m}}_{\varphi_{k},\left(N\right)}}{\partial {\hat{m}}_{P(\Theta ),(N-1)}}\\ \frac{\partial {\hat{m}}_{\varphi_{k},\left(N\right)}}{\partial m_{\varphi_{k},i}}\\ \frac{\partial {\hat{m}}_{\varphi_{k},\left(N\right)}}{\partial \sum_{C\cap D=\varphi_{k}}m_{B,(N-1)}m_{C,i}} \end{array}\right]\cdot {\left[\begin{array}{c} \frac{\partial m_{P(\Theta ),i}}{\partial \delta_{i}}\\ \frac{\partial {\hat{m}}_{\varphi_{k},\left(N-1\right)}}{\partial \delta_{i}}\\ \frac{\partial {\hat{m}}_{P(\Theta ),(N-1)}}{\partial \delta_{i}}\\ \frac{\partial m_{\varphi_{k},i}}{\partial \delta_{i}}\\ \frac{\partial \sum_{C\cap D=\varphi_{k}}m_{B,(N-1)}m_{C,i}}{\partial \delta_{i}} \end{array}\right]}^{T}\\ =\left[\begin{array}{c} {\hat{m}}_{\varphi_{k},\left(N-1\right)}\\ m_{P(\Theta ),i}\\ m_{\varphi_{k},i}\\ {\hat{m}}_{P(\Theta ),(N-1)}\\ 1 \end{array}\right]\cdot {\left[\begin{array}{c} -{\bar{w}}_{i}\\ 0\\ 0\\ {\bar{w}}_{i}\beta_{\varphi_{k},i}\\ \sum_{C\cap D=\varphi_{k}}m_{B,(N-1)}{\bar{w}}_{i}\beta_{\varphi_{k},i} \end{array}\right]}^{T} \end{array}$$

Equation (48) is derived using the chain rule, where the output confidence $\beta_{\varphi_{k},\left(N\right)}$ depends on the basic probability mass ${\hat{m}}_{\varphi_{k},\left(N\right)}$ and ${\hat{m}}_{P(\Theta ),(N)}$, which are further influenced by the CHCC parameter $\delta_{i}$. Therefore, the sensitivity is decomposed into the contributions of these intermediate variables. The term $\frac{\partial {\hat{m}}_{\varphi_{k},\left(N\right)}}{\partial \delta_{i}}$ captures how the combined mass assigned to health grade $\varphi_{k}$ changes with respect to $\delta_{i}$. Since the ER-rule fusion is recursive, this derivative is further decomposed into contributions from the previous fusion step and the current evidence source.

(55) $$\begin{array}{c} \frac{\partial {\hat{m}}_{P(\Theta ),(N)}}{\partial \delta_{i}}=\frac{\partial \prod_{\iota =1}^{N}\left(1-{\bar{w}}_{\iota }\delta_{\iota }\right)}{\partial \delta_{i}}\\ =-{\bar{w}}_{i}\prod_{\iota =1}^{N-1}\left(1-{\bar{w}}_{\iota }\delta_{\iota }\right) \end{array}$$

This term represents the sensitivity of the basic probability mass of power sets ${\hat{m}}_{P(\Theta ),(N)}$ with respect to $\delta_{i}$. Indicating that the impact of $\delta_{i}$ propagates multiplicatively through all fused evidence.

Therefore, the sensitivity of CHCC to the confidence level $\beta_{\varphi_{k},\left(N\right)}\left(t\right)$ can be determined by Equation (51).

(56) $$\xi \left(t\right)=\sum_{i=1}^{N}\sum_{k=1}^{M}\frac{\partial \beta_{\varphi_{k},\left(N\right)}\left(t\right)}{\partial \delta_{i}}$$

In practical engineering fields, ξ is a sensitivity factor of the output confidence to the CHCC, indicating the validity of the subsystem’s causality-informed correlation in the model output.

Based on the above analysis, we can further derive the sensitivity factor of output utility η(t) with respect to $\eta \left(t\right)=\sum_{\varphi_{k}\subseteq \Theta }\eta \left(\varphi_{k}\right)\beta_{\varphi_{k},\left(N\right)}\left(t\right)$, define the output utility sensitivity factor $\xi^{\left(\eta \right)}$ as

(57) $$\xi^{\left(\eta \right)}=\left|\frac{\partial \eta }{\partial \delta_{i}}\cdot \frac{\delta_{i}}{\eta }\right|$$

Based on Equation (52), we can determine the tolerance range for CHCC. Specifically, factors such as external disturbances can cause fluctuations in the calculated value of $\delta_{i}$, which in turn affect the output utility η(t). In engineering practice, a tolerance coefficient ρ is typically defined to represent the maximum acceptable range of fluctuation in the output utility, as shown in Equation (53).

(58) $$\left|\Delta \eta \right|⩽\rho \eta$$

where $\left|\Delta \eta \right|$ represents the range of fluctuations in output utility. The specific value of the tolerance coefficient ρ is determined based on the actual equipment and operating conditions.

Under conditions of small disturbances

(59) $$\Delta \eta \approx \frac{\partial \eta }{\partial \delta_{i}}\cdot \Delta \delta_{i}$$

Therefore, as long as Equation (55) is satisfied, the fluctuations in output utility will not exceed ρη.

(60) $$\left|\frac{\partial \eta }{\partial \delta_{i}}\cdot \Delta \delta_{i}\right|⩽\rho \eta$$

This gives the upper bound of the fluctuation of $\delta_{i}$

(61) $$\left|\Delta \delta_{i}\right|⩽\frac{\rho \eta }{\left|\frac{\partial \eta }{\partial \delta_{i}}\right|}=\frac{\rho \delta_{i}}{\xi^{\left(\eta \right)}}$$

In summary, the tolerance range for $\delta_{i}$ is

(62) $${\tilde{\delta }}_{i}\in \left[{\delta_{i}}^{\left(\mathrm{up}\right)},{\delta_{i}}^{\left(\mathrm{low}\right)}\right]=\left[\delta_{i}-\frac{\rho \delta_{i}}{\xi^{\left(\eta \right)}},\delta_{i}+\frac{\rho \delta_{i}}{\xi^{\left(\eta \right)}}\right]\cap \left[0,1\right]$$

where ${\tilde{\delta }}_{i}$ represents the disturbed CHCC of the subsystem $X_{i}$. □

### 4.2. Robustness Analysis

The analysis of model robustness focuses primarily on scenarios where the conditions for the subsystem are outlined in Section 3.2 are not met. Condition ① is relatively common in system design and will not be discussed in detail here.

(1) Robustness analysis for Condition ②

First, analyze the scenario where Condition ② is not met, i.e., the case where “No closed-loop feedback structures exist between subsystems” is not satisfied. First, according to the research by Butler [36] and Quian [47], when there is a feedback loop between systems $X_{i}$ and $X_{j}$, the driving relationships between $X_{i}$ and $X_{j}$ become mutually coupled and difficult to distinguish, rendering causal analysis of $X_{i}$ and $X_{j}$ ineffective. Current approaches use non-system dynamics methods (such as structural equation modeling (SEM) [48]) to construct cyclical causal graphs, thereby enabling causal analysis that accounts for closed-loop feedback. However, constructing cyclical causal graphs requires long-term, stable monitoring data to yield accurate results, which is difficult to achieve for complex equipment with variable operating conditions and inherent performance degradation.

**Figure 3:**
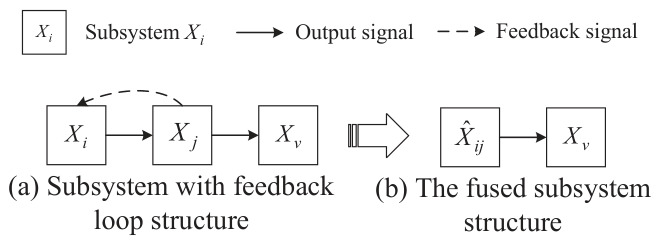
The handling method for Condition ②.

For cases where Condition ② is not met, the following solution is provided. In the presence of a feedback loop between subsystems $X_{i}$ and $X_{j}$, they are consolidated into a single subsystem $\hat{X}ij$, from which a subsystem-level health indicator $\hat{\phi }ij$ is derived. Additionally, ${\hat{X}}_{ij}$ includes all the underlying indicators $h_{i}$ and $h_{j}$ of $X_{i}$ and $X_{j}$. The above processing procedure is shown in Figure 3.

From the perspective of model robustness, treating $X_{i}$ and $X_{j}$ as a single subsystem ${\hat{X}}_{ij}$ introduces the risk of erroneous causal relationship judgments, indicating uncertainty in the causal structure of the subsystem. Specifically, the aforementioned operation will inevitably lead to changes in the correlation matrix K:

(63) $$K=\left[\begin{array}{ccc} K(Y_{i},Y_{i}) & & \\ K(Y_{j},Y_{i}) & K(Y_{j},Y_{j}) & \\ K(Y_{v},Y_{i}) & K(Y_{v},Y_{j}) & K(Y_{v},Y_{v}) \end{array}\right]\Rightarrow \hat{K}=\left[\begin{array}{cc} K({\hat{Y}}_{ij},{\hat{Y}}_{ij}) & \\ K(Y_{v},{\hat{Y}}_{ij}) & K(Y_{v},Y_{v}) \end{array}\right]$$

where $\hat{K}$ indicates the transformed correlation matrix. ${\hat{Y}}_{ij}$ denote the output signal of subsystem ${\hat{X}}_{ij}$, and ${\hat{Y}}_{ij}=Y_{j}$. Therefore, $\hat{K}$ can be equivalently expressed as:

(64) $$\hat{K}=\left[\begin{array}{cc} K(Y_{j},Y_{j}) & \\ K(Y_{v},Y_{j}) & K(Y_{v},Y_{v}) \end{array}\right]$$

According to Equations (22) and (23), the CHCC values before and after transformation can be calculated separately from K and $\hat{K}$, respectively. Then the CHCC variation can be obtained, denoted as $\Delta \delta_{i}$. However, according to Equations (54) and (55), it can be deduced that

(65) $$\Delta \eta ⩽\left|\frac{\partial \eta }{\partial \delta_{i}}\right|\cdot \Delta \delta_{i}$$

Equation (60) indicates that the output utility variation Δη caused by $\Delta \delta_{i}$ is bounded. Even if causal directions are partially misidentified, their effect is reflected as perturbations in the CHCC values. Therefore, robustness to CHCC variation implies robustness to structural uncertainty.

Based on the tolerance range of CHCC obtained in Section 4.1, it can be known that when using the ERr-CIC model to assess the health state of complex equipment with feedback loops in subsystems, the solution proposed in this subsection can be adopted to calculate the value of $\Delta \delta_{i}$. If $\Delta \delta_{i}$ is within the tolerance range, the output utility obtained by the model can be adopted.

(2) Robustness analysis for Condition ③

The CCM-based causal inference framework assumes that external inputs remain constant. However, in real-world engineering systems, subsystems are often subject to time-varying external control signals. To address this problem, the external control signal is used to control the device to perform periodic and stable actions. In this case, the control signal can be regarded as an unchanged external excitation. According to CCM theory, such deterministic inputs can be incorporated into the reconstructed state space without destroying the underlying manifold structure.

From the perspective of robustness, periodic external control signals introduce structured and bounded perturbations, rather than stochastic disturbances. These perturbations do not alter the intrinsic coupling relationships among subsystems. Therefore, the inferred causal relationships remain stable under such conditions.

## 5. Case Study

### 5.1. Experiment Setting

As a reliable and precise measuring device, the electronic theodolite is widely used in hydraulic engineering, resource exploration, and military fields. The PAMD, serving as the core component of the electronic theodolite, is tasked with the measurement, computation, and display of angles. Therefore, assessing the health state of the PAMD is of significant importance. A specific model of PAMD was selected for the experimental analysis.

The PAMD consists of three main subsystems [49,50]: a photoelectric conversion unit (subsystem 1), a differential amplification unit (subsystem 2), and a microprocessor (subsystem 3). The photoelectric conversion unit converts received optical signals into electrical signals that encode angular information. These signals are then passed to the differential amplification unit, which performs filtering, transformation, and amplification. The processed signals are subsequently fed into the microprocessor, which includes a microcontroller unit (MCU) and peripheral circuits. The MCU executes computational algorithms to determine the theodolite’s rotation angle and displays the measurement results.

However, PAMDs are typically enclosed in electronic theodolites with high precision. It is difficult to obtain the output signal from the PAMD within an electronic theodolite without damaging its internal optical structure. Therefore, the experiment employed simulated experimental equipment that shares the same subsystems, working mechanism, and dynamic coupling relationships as the actual PAMD.

The system dynamics equations of the simulation equipment were established with reference to previous studies on the dynamic modeling of theodolite systems [51,52] to ensure that the coupling relationships among the simulated subsystems were consistent with the main dynamic interaction characteristics of the actual PAMD. The dynamic equations for the simulation system were set up as follows:

(66) $$\left\{\begin{array}{c} Y_{1}\left(t+1\right)=a_{1}Y_{1}\left(t\right)+b_{1}G\left(\theta \left(t\right),L\left(t\right)\right)+c_{1}+w_{1}\left(t\right)\\ Y_{2}\left(t+1\right)=a_{2}Y_{2}\left(t\right)+b_{2}Y_{1}\left(t\right)+c_{2}+w_{2}\left(t\right)\\ Y_{3}\left(t+1\right)=a_{3}Y_{3}\left(t\right)+b_{3}\phi \left(Y_{2}\left(t\right)\right)+c_{3}+w_{3}\left(t\right) \end{array}\right.$$

where $Y_{i}$ denotes the output of subsystem $X_{i}$ at time *t*; $a_{i},i\in \left[1,3\right]$ represents the dynamic retention coefficient; $c_{i}$ is the bias term; $b_{i}$ denotes the gain term; L(t) represents the incident light intensity, and G(·) indicates the optical signal modulation function; ϕ(·) denotes the calibrated angular solution function; and $w_{i}$ represents the noise term, which accounts for potential electronic fluctuations in the actual equipment as well as measurement uncertainties. The noise term was set to Gaussian white noise $w_{i}∼N\left(0,{\sigma_{i}}^{2}\right)$ in this paper to avoid systematic bias and minimize the possibility of introducing correlations other than those caused by causal coupling. The variance $\sigma_{i}$ was set to $\sigma_{i}=\hbar_{i}\cdot {\bar{Y}}_{i}$, where $\hbar_{i}$ represents the noise ratio coefficient and ${\bar{Y}}_{i}$ represents the mean of the subsystem’s output time series.

By analyzing the dynamic equations of the simulated equipment system, it can be concluded that the formulated equations capture the key causal interactions among subsystems, including optical signal modulation, inter-subsystem coupling, and nonlinear transformation processes. The parameters $a_{i}$ and $b_{i}$ characterize the intrinsic dynamic retention and coupling strength, respectively, while the functions G(·) and ϕ(·) represent the physical transformation mechanisms corresponding to optical modulation and angular solution processes in the actual equipment. Therefore, the model retains the core dynamic behaviors and interaction pathways observed in real PAMD equipment.

**Figure 4:**
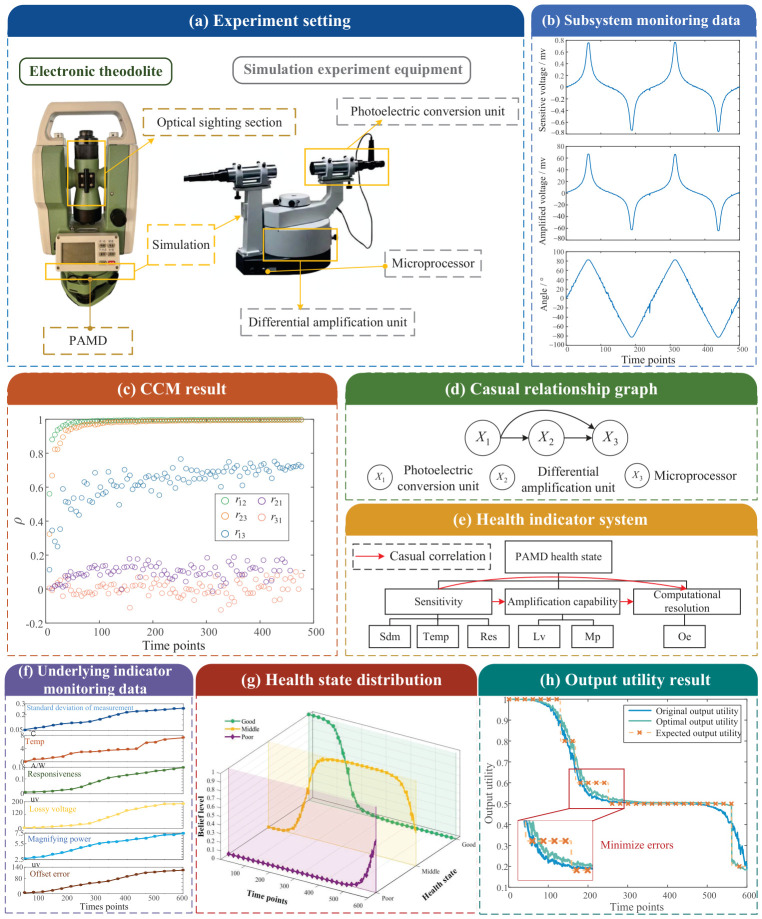
Experimental procedure.

The position of the PAMD in the electronic theodolite and the composition of the simulation experimental equipment are shown in Figure 4a.

### 5.2. Causal Relationships Inference

Section 3.2 of this paper discusses the applicability conditions of complex equipment causal analysis. Through mechanism analysis of PAMD, it is evident that it meets conditions ① and ②: Each subsystem of PAMD has only one output, and the subsystems are in an open-loop structure with no feedback elements. To satisfy condition ③, this paper periodically collects the output of the subsystem, so that the external input can be regarded as a constant “0”.

Using the subsystem output time series as the projection of the subsystem state space in one-dimensional space. The output signals are directly acquired from the built-in output ports of the simulated experimental platform. This acquisition mechanism ensures that the recorded data faithfully reflects the intrinsic evolution of each subsystem, avoiding additional measurement noise or distortion introduced by external sensing processes. Periodically collect the outputs of each subsystem, collecting a total of two cycles as shown in Figure 4b. The outputs of subsystems 1, 2, and 3 are set as $Y_{1}=\left\{Y_{1}\left(t\right)\left|t\in [1,500]\right.\right\}$, $Y_{2}$, and $Y_{3}$, respectively.

According to the average mutual information method and the false nearest neighbors method, the embedding dimension and delay time are determined as m=4, τ=5. The reconstructed state space is represented as $M_{1}$, $M_{2}$, and $M_{3}$. It is worth noting that the choice of two operational cycles represents a trade-off between data sufficiency and computational efficiency. Empirically, this length is adequate to capture the dominant dynamic patterns of the system while avoiding redundancy and excessive computational overhead in later processing stages. To verify whether the subsystem output data obtained from monitoring can be used for causal analysis, AF and RF metrics of $M_{1}$, $M_{2}$, and $M_{3}$ are validated. The test results are shown in Table 1. It can be seen that the three state spaces of the reconstruction, AF and RF values, all approach 1, meeting the requirements.

Using CCM to infer the causal relationships of subsystems, the results are shown in Figure 4c. From the figure, it can be seen that $r_{12}$ and $r_{23}$ quickly converge to “1” when the sample size increases to around 50. $r_{13}$ begins to converge when the sample size increases to around 150. $r_{21}$ and $r_{31}$, however, fluctuate around “0” and do not converge. In summary, the causal relationship among subsystems 1, 2, and 3 is: $X_{1}\to X_{2}$, $X_{1}\to X_{3}$, $X_{2}\to X_{3}$. The causal graph is shown in Figure 4d.

### 5.3. Health-State Assessment

PAMD’s health indicator system is constructed as follows [53,54]: ① Primary indicator: health state of PAMD. ② Subsystem-level indicators: Based on the operational mechanism analysis, primary functional assessment, and expert knowledge, the secondary indicators of the PAMD are determined as: sensitivity, amplification capability, and computational resolution. ③ Underlying indicators: according to the knowledge of experts and product specifications, six underlying indicators are selected: standard deviation of measurement (sdm), temperature (temp), responsiveness (res), lossy voltage (lv), magnifying power (mp), and offset error (oe). The health indicator system is shown in Figure 4e. Based on GB/T 5080 [55] and product instructions, PAMD health state is categorized into the following three health grades:

(67) $$\Theta =\left\{\varphi_{1},\varphi_{2},\varphi_{3}\right\}=\left\{Good,Middle,Poor\right\}$$

By varying the internal state parameters of the simulation equipment, the system was able to generate monitoring data ranging from healthy to poor health grades. The simulation device was internally equipped with a Hall effect sensor to measure current and voltage values at a measurement frequency of 100–250 kHz, and a temperature sensor with a measurement frequency of 100 Hz. Monitoring data of underlying health indicators were collected using the built-in sensors of the simulation equipment (Figure 4f). Sampling was performed based on the actual duration of a single electronic theodolite measurement. Since different sensors had varying sampling frequencies, the monitoring data in Figure 4f were first aligned on the timeline, resulting in a total of 600 time points. As can be seen from Figure 4f, the indicators deviated from the healthy values to varying degrees as the duration of use increased.

According to GB/T 36537-2018 [56], the reference values of each underlying health indicator are obtained as Table 2.

According to the indicator reference value and Equation (24), the indicator test data can be transformed into the form of a confidence level. The CHCC for evidence of subsystem-level indicators 1, 2, and 3 were calculated using Equations (14)–(23), with the results as: $\delta_{1}=1,\delta_{2}=0.5067,\delta_{3}=0.3619$. It can be seen that subsystem-level indicator 1 is independent of the other subsystem-level health indicators. Based on the signaling sequence between the subsystems, the fusion order of subsystem 1, 2, and 3 can be determined as: SeqF(1)=1,SeqF(2)=2,SeqF(3)=3.

According to the ERr-CIC model, the health state of the PAMD is obtained as Figure 4g. The curves in the graph are color-coded, with green, yellow, and purple representing Good, Middle, and Poor health states, respectively. The overall health state of PAMD demonstrates a gradual decline over time, consistent with the expected performance degradation of the equipment.

According to Equation (43), the original output utility of PAMD is shown in Figure 4h. Industry experts determine the expected output utility based on PAMD’s actual operational scenarios and in conjunction with standard GB/T 37084-2018 [57]. It can be seen that the original output utility basically follows the same trend as the expected output utility. The root mean square error (RMSE) between the original output and the expected output is 0.056. However, during the transition period of changes in health state, the deviations are generally larger. Optimize the original ERr-CIC model according to the objective function and constraints shown in Equations (44)–(47). The ERr-CIC model utilized the sequential quadratic programming (SQP) algorithm, with a maximum number of iterations set to $1\times 10^{3}$ and the optimization terminates when the step size falls below $1\times 10^{-6}$. The parameter optimization results are shown in Table 3. The RMSE of the optimized model is 0.032. RMSE decreases from 0.056 to 0.032 after optimization, corresponding to a relative reduction of 75%.

### 5.4. Numerical Validation of Model Performance

This subsection conducts numerical verification of the sensitivity and robustness analysis result presented in Section 4, and specifically analyzes the practical application value of sensitivity factors in the health-state assessment of complex equipment.

**Figure 5:**
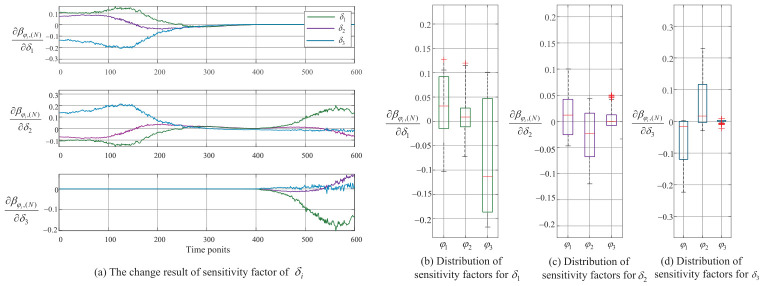
Calculation results of sensitive factors.

First, the sensitivity factor is calculated based on the health-state assessment results from Section 5.2, as shown in Figure 5.

Figure 5a shows the sensitivity analysis results of confidence levels for various health grades on $\delta_{i}$. The greater the result, the more sensitive the change in health grade confidence level $\beta_{\varphi_{k},\left(N\right)}$ to $\delta_{i}$. Figure 5a indicates that each health state exhibits a certain degree of sensitivity to $\delta_{1}$, particularly when the complex equipment is at health grade $\varphi_{3}$. To further clarify the impact of $\delta_{i}$ on the health-state assessment results, the distribution of sensitive factor results is presented in the form of a box plot, as shown in Figure 5b. For each $\delta_{i}$, a longer length of the box plot indicates a greater impact on the confidence level of that health grade. As can be seen more clearly from Figure 5b, $\delta_{1}$ has the greatest impact on the health-state assessment results, and especially for those with a health grade of Poor. This further demonstrates the conclusion from Section 4.1 that subsystems with larger $\delta_{i}$ values have a greater impact on the health-state assessment results. Since $\delta_{2}$ and $\delta_{3}$ are numerically close, their sensitivity analysis results are also relatively close. However, Figure 5b still shows that the assessment results are more sensitive to $\delta_{2}$ than to $\delta_{3}$.

**Figure 6:**
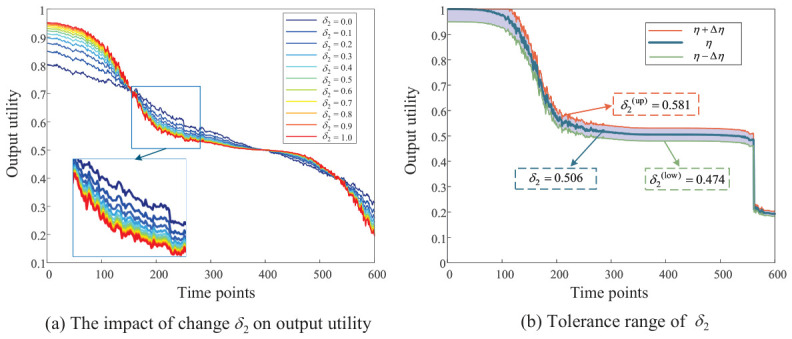
CHCC tolerance range analysis.

Furthermore, the tolerance range of sensitive factors was calculated, and the results are shown in Figure 6.

Since the tolerance range analysis step is identical for all $\delta_{i}$, we use $\delta_{2}$ as an example for analysis. Firstly, change the value of $\delta_{2}$ by 0.1 steps to analyze its impact on the output utility, as shown in Figure 6a. As can be seen from Figure 6a, the impact of changes in $\delta_{2}$ on output utility is not proportional. Assuming the tolerance coefficient ρ=5%, the tolerance range of $\delta_{2}$ can be obtained as ${\tilde{\delta }}_{2}\in \left[0.474,0.581\right]$ according to Equation (57), as shown in Figure 6b. This result provides a clear quantitative guideline for parameter selection in practical deployment.

From a robustness perspective, this nonlinearity implies that small perturbations in $\delta_{2}$ do not lead to significant fluctuations in the output, reflecting the stability of the ERr-CIC model structure. In particular, within certain intervals, the output utility changes smoothly, suggesting the existence of locally insensitive regions where the model maintains consistent performance despite parameter disturbances. The tolerance range shown in Figure 6b demonstrates that within a certain variation range of $\delta_{2}$, the deviation of the output utility remains within an acceptable threshold. In other words, the model output is insensitive to moderate perturbations of $\delta_{2}$, further verifying the robustness of the model.

## 6. Comparison Experiment

To further illustrate the feasibility and superiority of the method proposed in the article, this paper conducts comparative experiments from two aspects: the comparison of causal inference methods and the health-state assessment methods.

### 6.1. Comparison of Causal Inference Methods

To justify the use of the CCM method, this section compares it with two mainstream approaches for causal inference in nonlinear dynamic systems: transfer entropy (TE) and nonlinear Granger (NG).

The TF of $X_{i}\to X_{j}\left(i,j\in \left[1,3\right]\right)$ is defined as

(68) $$TE_{X_{i}\to X_{j}}=\sum p(Y_{i}\left(t+1\right),{Y_{i}}^{\left(l\right)},{Y_{j}}^{\left(k\right)})\log\frac{p(Y_{i}\left(t+1\right)\left|{Y_{i}}^{\left(l\right)},\right.{Y_{j}}^{\left(k\right)})}{p(Y_{i}\left(t+1\right)\left|{Y_{i}}^{\left(l\right)}\right.)}$$

where ${Y_{i}}^{\left(l\right)}=\left(Y_{i}\left(t\right),Y_{i}\left(t-1\right),\ldots ,Y_{i}\left(t-l+1\right)\right)$,${Y_{j}}^{\left(k\right)}=\left(Y_{j}\left(t\right),Y_{j}\left(t-1\right),\ldots ,Y_{j}\left(t-k+1\right)\right)$ represent the output data of the subsystems $X_{i}$ and $X_{j}$ with lengths *l* and *k*, respectively. In this study, l=k=1 was adopted to ensure robust probability estimation under limited samples and to provide a fair baseline comparison with CCM. The joint and conditional probabilities were estimated using a histogram-based discretization method. When $TE_{X_{i}\to X_{j}}>0$ occurs, then $X_{i}\to X_{j}$ holds.

For the NG method, a restricted model and an unrestricted model were constructed:

(69) $$Y_{j}\left(t+1\right)=f\left(Y_{j}\left(t\right),Y_{j}\left(t-1\right),\ldots ,Y_{j}\left(t-b+1\right)\right)+{\varepsilon_{t}}^{\left(r\right)}$$

(70) $$Y_{j}\left(t+1\right)=g\left(Y_{j}\left(t\right),Y_{j}\left(t-1\right),\ldots ,Y_{j}\left(t-b+1\right),Y_{i}\left(t\right),Y_{i}\left(t-1\right),\ldots ,Y_{i}\left(t-b+1\right)\right)+{\varepsilon_{t}}^{\left(u\right)}$$

where *b* represents the lag order. In this study, the lag order was set to b=2 for all subsystem pairs to balance predictive capability and estimation stability under the limited sample size. $f\left(\cdot \right):R^{p}\to R,g\left(\cdot \right):R^{2p}\to R$ are nonlinear prediction functions. ${\varepsilon_{t}}^{\left(r\right)}$, ${\varepsilon_{t}}^{\left(u\right)}$ are the residuals of the two models, respectively. In this study, Gaussian-kernel nonlinear regression was used to implement both models.

(71) $$GC_{X_{i}\to X_{j}}=\ln\frac{Var({\varepsilon_{t}}^{\left(r\right)})}{Var({\varepsilon_{t}}^{\left(u\right)})}$$

$GC_{X_{i}\to X_{j}}>0$ indicates that the addition of the historical information of $X_{i}$ reduces the prediction error of $Y_{j}\left(t+1\right)$, implying $X_{i}\to X_{j}$ holds. To evaluate statistical significance, a permutation test was further performed by randomly shuffling the source series and recalculating the causality statistic to generate the null distribution.

**Figure 7:**
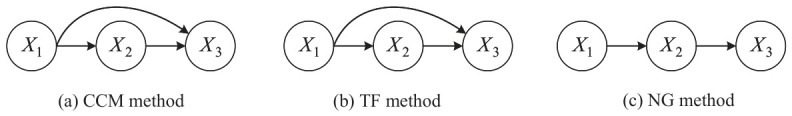
Causal relationship inference results.

The causal relationship graph obtained by methods CCM, TF, and NG is shown in Figure 7.

As shown in Figure 7, CCM and TF produce the same causal graph, whereas the NG method misses the edge from $X_{1}$ to $X_{3}$. From the PAMD working mechanism, the three subsystems operate in a serial signal-processing chain and are dynamically coupled. In such a nonlinear coupled system, the state information of upstream subsystems can propagate through intermediate subsystems and remain embedded in the dynamics of downstream subsystems. In this sense, the edge $X_{1}\to X_{3}$ is physically and dynamically reasonable. In summary, from the perspectives of consistency in results and mechanistic analysis, the causal relationship graph obtained using the CCM and TF methods is more reliable.

The three methods were then compared in terms of computational efficiency and the accuracy of health-state assessments. Computational efficiency is defined as the runtime of each method, recorded over 20 independent runs, with the mean and variance of runtime subsequently calculated. Health-state assessment accuracy is compared using the following process: First, the CHCC is calculated separately for each causal relationship graph derived from each method. Second, the CHCC is input into the ERr-CIC model, and health-state assessment is performed using the underlying indicator monitoring data obtained in Section 5.2, following the same model optimization process. Finally, the RMSE between the model’s output utility and the expected output utility is compared.

The comparison results are shown in Table 4.

The NG method achieved the lowest accuracy in health-state assessment, with an RMSE of 0.078. The primary reason for this may be that discrepancies in the causal relationship graph analysis led to errors in the CHCC calculation, which in turn affected the accuracy of the health-state assessment results. At the same time, the accuracy of the health-state assessment results further demonstrates the rationality of the CCM method and the TF method in analyzing causal relationship graphs. Although CCM and TF methods have the same assessment accuracy, the TF method requires probability density estimation, resulting in significantly lower computational efficiency compared to CCM. In summary, the CCM is suitable for the causal inference of nonlinear dynamical systems such as complex equipment.

### 6.2. Comparison of Health-State Assessment Methods

Comparison models include: ① CNN-Transformer model [58]. This model uses a CNN network to achieve end-to-end feature extraction between health indicator monitoring data and output utility, and then utilizes the attention mechanism of the Transformer network to deeply capture nonlinear relationships, such as correlations between features, achieving health-state assessment of complex equipment. ② Graph Convolution Network (GCN) model [59]. Compared with traditional deep learning methods, this model can learn from graphs, a non-matrix-based information representation method, which is suitable for capturing the complex causal relationship features from the subsystem causal relationship graph. ③ T-S fuzzy model [60]. The T-S model, as a typical semi-quantitative information method, can effectively utilize quantitative monitoring data and qualitative knowledge and convert them into fuzzy rules to achieve complex equipment health-state assessment. The hyperparameters of the comparison models are shown in Table 5.

**Figure 8:**
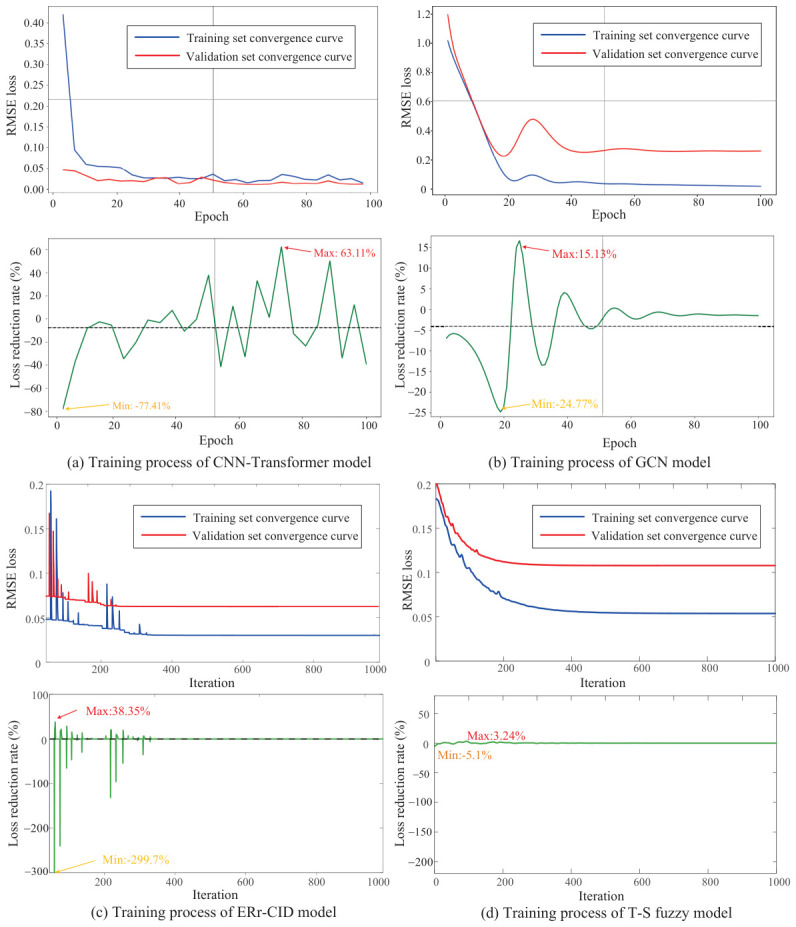
Model training and optimization process. The dashed line in the figure represents the reference line for the variation of the loss reduction rate.

To ensure a fair comparison, all models were constructed using the same input-output setting. Specifically, the input of all models consisted of the same six underlying indicators as shown in Table 3, while the output was the same output utility η(t). All models used the same training and validation sets. Since the compared models belong to different methodological categories, their parameters were optimized using the solvers commonly adopted for each type of model. The CNN-Transformer and GCN models employed the Adam optimizer with a learning rate of $1\times 10^{-3}$, which is reduced by a factor of 0.5 every 10 epochs. The momentum parameter is set to 0.9, and L2 regularization with a weight decay of $1\times 10^{-4}$ is applied to prevent overfitting. The T-S fuzzy model used the same SQP optimization algorithm as the ER model, with identical optimization parameter settings: the maximum number of iterations was set to $1\times 10^{3}$ and the optimization terminates when the step size falls below $1\times 10^{-6}$. The model convergence curve and training loss rate are shown in Figure 8.

Firstly, the CNN-Transformer model exhibits a rapid decrease in error during the initial training phase, indicating its strong ability in feature extraction and fitting. However, there is a certain degree of oscillation in the middle and later stages, with a more pronounced fluctuation in the loss reduction rate, suggesting that the stability of its optimization process needs improvement. Secondly, the ERr-CIC model converges quickly overall, with a small gap between training and validation errors, demonstrating good generalization ability. Its loss reduction process is relatively smooth, with only slight fluctuations in the early stages, and then quickly stabilizes, indicating that the model optimization process is efficient and stable. For the GCN model, although there is a significant decrease in error during the initial training phase, the overall fluctuation is large, especially in the loss reduction rate curve, which shows frequent oscillations. This suggests that the model is susceptible to gradient fluctuations during the optimization process and has relatively weak stability. Finally, the T-S fuzzy model demonstrates the smoothest and most stable convergence process. Its training and validation errors decrease uniformly and quickly stabilize, with minimal fluctuation in the loss reduction rate, indicating that the model has good robustness and convergence performance during the optimization process. In summary, compared to other models, the T-S fuzzy model performs optimally in terms of convergence speed and stability, while the ERr-CIC model has certain advantages in terms of generalization ability; the CNN-Transformer and GCN models, although possessing strong fitting capabilities, still have room for improvement in training stability.

**Figure 9:**
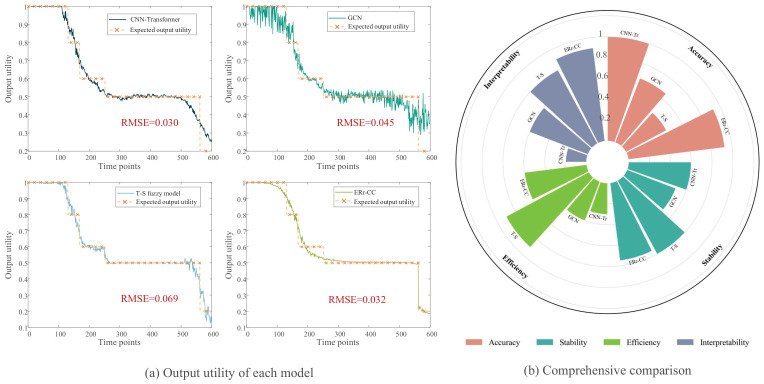
Model comparison.

The comparison of model output performance results is shown in Figure 9a. It can be seen that among the four models, the CNN-Transformer has the highest accuracy, with an RMSE of 0.030. The reason the GCN model, also as a deep learning method, performs poorly in terms of accuracy may be that it does not adequately capture the relationship between causal coupling in system dynamics and statistical correlations, resulting in biased evaluations.

**Figure 10:**
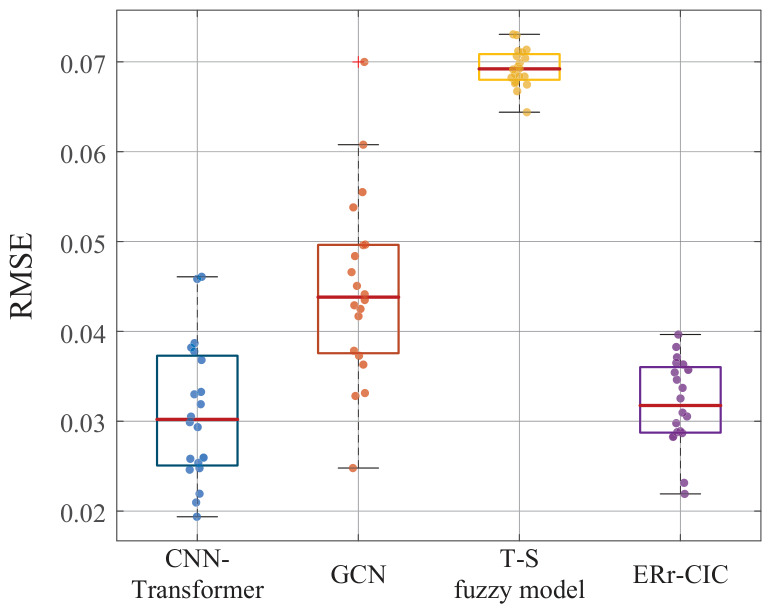
Distribution of model results from 20 trials. The blue, orange, yellow, and purple dots represent the distribution of RMSE results for CNN Transformer, GCN, T-S fuzzy model, and ERr CIC, respectively.

To verify whether the differences in model performance are statistically significant and to minimize performance fluctuations caused by random factors, this paper conducts a Friedman test on the model results. The specific steps are as follows: ① The monitoring data obtained in subsection A are randomly split into training and validation sets over 20 independent trials. ② In each trial, the model uses the same training and validation sets. ③ Record the mean and standard deviation of RMSE for each trial. The distribution of the model results is shown in Figure 10. It can be seen that the ERr-CIC model and the T-S fuzzy model produce more stable results. The statistics of 20 trials for each model are shown in Table 6.

Based on the statistical measures derived from the above results, a Friedman test was conducted. Specifically, the models were first ranked according to their RMSE values from each run, with lower RMSE values corresponding to higher rankings. Subsequently, the average rank for each model was calculated as shown in Equation (67).

(72) $${\bar{R}}_{j}=\frac{1}{N}\sum_{i=1}^{N}r_{ij}$$

where $r_{ij}$ represents the ranking of model *j* in its *i*th run. ${\bar{R}}_{j}$ indicates the average rank of the model *j*. The Friedman statistic is expressed as:

(73) $${\chi^{2}}_{F}=\frac{12N}{k(k+1)}\sum_{j=1}^{k}{{\bar{R}}_{j}}^{2}-3N\left(k+1\right)$$

where N=20 denotes the number of runs. k=4 represents the number of models. ${\chi^{2}}_{F}$ approximately follows a chi-squared distribution with k−1 degrees of freedom. After calculation, ${\chi^{2}}_{F}=10.8$, there is: $p=P({\chi^{2}}_{3}⩾10.8)=0.0128$. In summary, the Friedman test indicates that the differences in RMSE among the compared models are statistically significant.

However, model performance cannot be fully assessed based solely on accuracy. Below, we conduct a comprehensive evaluation of each model, including interpretability, stability, efficiency, and accuracy four comparison metrics. ① Model accuracy is determined by the average RMSE of the results from 20 runs. ② Model stability is determined by the variance of the RMSE across 20 model runs. ③ Efficiency is assessed by the average time over 20 runs. ④ Model interpretability is assessed by 5 experts based on the traceability of output, and the comprehensibility of results. Each model was evaluated using the final converged parameters obtained after optimization. The results of the model comparison are shown in Table 7.

To make the comparison results clearer, each result has been mapped to the 0–1 range, as shown in Figure 9b. While ERr-CIC exhibits lower model accuracy compared to CNN-Transformer, it significantly outperforms it in terms of interpretability and efficiency. When making maintenance decisions, methods with high interpretability are preferred. At the same time, although the classic T-S model performs the worst in terms of model accuracy, it has advantages in efficiency and interpretability. Overall, the ERr-CIC model performs relatively evenly in all aspects, making it more suitable for assessing the health state of complex equipment with causality-informed correlation in engineering practice.

## 7. Conclusions

In this paper, a health-state assessment model for complex equipment is developed based on evidential reasoning rules. Two main problems are addressed. The first is that the health-state assessment model does not consider the causality-informed correlation among health indicators and the fusion order of indicators. The second is the sensitivity analysis of the performance of the health state model. The main innovations can be categorized into the following four points:

- Aiming at the problem that current health-state assessment models do not consider the causality-informed correlation of subsystem-level indicators, the CCM is used to determine causal coupling relationships, followed by calculating the CHCC to quantify the magnitude of causality-informed correlation.
- To address the problem that current health-state assessment models do not consider the fusion order of indicators, this paper presents a method to determine the fusion order based on signaling sequences.
- This study contributes to the development of a health-state assessment model utilizing ERr-CIC, which integrates subsystem causality-informed correlation into the model parameters, providing a possible approach to addressing the complexity of equipment health-state assessments.
- The lack of performance analysis of assessment models in current complex equipment health-state assessment methods is addressed. In this paper, the sensitivity of the output results to the CHCC for the proposed ERr-CIC model is analyzed. Based on actual engineering requirements, the tolerance range of the CHCC is further obtained. The sensitivity analysis conclusion is validated through experiments in the case study.

However, the ERr-CIC model still has the following limitations:

- The model assumes that the underlying indicators of the subsystems are independent of each other. However, in some cases, correlations may also exist between underlying indicators. Such correlations can lead to redundant health information as well, resulting in biased health-state assessment results. In the future, further research will be conducted on the correlations between underlying indicators of subsystems, and a complete correlation processing system will be established from underlying indicators to the subsystem level.
- There are three conditions that need to be met when conducting subsystem causal analysis in the current model. When these conditions are not met, the results of causal analysis may be affected, which in turn affects the accuracy of the CHCC. Extending the causal analysis method of the ERr-CIC model to cyclic causal diagrams to enhance the applicability of the model in different scenarios is one of the important research directions for the future.
- The validation in this study is conducted based on a mechanism-driven simulation model. Although the simulation is constructed from system dynamics equations and measurement principles to approximate realistic operating conditions, validation on real-world equipment is still necessary. Future work will focus on applying the proposed method to practical engineering datasets to further demonstrate its applicability and robustness.

## Figures and Tables

**Table 1:** AF, RF metrics test results.

Reconstructed State Space	AF	RF
$M_{1}$	1	1
$M_{2}$	0.98	1
$M_{3}$	0.99	0.98

**Table 2:** Reference values of health indicators.

Health Indicator	$\varphi_{1}$	$\varphi_{2}$	$\varphi_{3}$
sdm	0.2	0.446	0.5
temp	20 °C	27.5 °C	30 °C
res	0.5 A/W	0.66 A/W	0.7 A/W
lv	100 uv	283.4 uv	325 uv
mp	100	107.171	110
oe	152.588 uv	271.727 uv	305.176 uv

**Table 3:** Model parameter optimization results.

Health Indicator	$\varphi_{1}$	$\varphi_{2}$	$\varphi_{3}$
sdm	0.215	0.450	0.680
temp	22.160 °C	27.520 °C	30.184 °C
res	0.498 A/W	0.667 A/W	0.741 A/W
lv	100.211 uv	283.423 uv	326.168 uv
mp	102.175	117.178	125.641
oe	152.591 uv	275.610 uv	308.200 uv
CHCC	0.983	0.506	0.361
utility value	1	0.505	0.169

**Table 4:** Comparison of computational efficiency and health-state assessment accuracy among causal inference methods.

Causal Inference Method	Average of Runtime	Variance of Runtime	Health-State Assessment Accuracy
CCM	0.84 s	0.05 s	0.032
TF	4.73 s	0.21 s	0.032
NG	1.92 s	0.14 s	0.078

**Table 5:** Model hyperparameters.

Model Hyperparameters	CNN-Transformer	GCN	T-S Fuzzy Model
input dimension	3	3	/
output dimension	1	1	/
number of attention heads	8	/	/
normalization method	/	symmetric normalization	/
batch size	16	16	/
number of rules	/	/	9
membership function	/	/	Gaussian membership function
de-blurring methods	/	/	weighted average

**Table 6:** Statistics of model results from 20 runs.

Model	Mean RMSE	Std	95% CI	Average Rank
CNN-Transformer	0.0304	0.0085	[0.0289, 0.0319]	1.9
GCN	0.0457	0.0098	[0.0447, 0.0467]	2.8
T-S fuzzy model	0.0692	0.0018	[0.0675, 0.0709]	3.1
ERr-CIC	0.0321	0.0045	[0.0314, 0.0328]	2.2

Note: Std represents the standard deviation of RMSE across 20 independent runs. The 95% CI denotes the confidence intervals of RMSE and was calculated based on the t-distribution.

**Table 7:** Comparison metrics result.

Comparison Metrics	CNN-Transformer	GCN	T-S Fuzzy Model	ERr-CIC
Accuracy	0.030	0.045	0.069	0.032
Stability	0.0085	0.0098	0.0018	0.0045
Efficiency	3.6 s	5.4 s	0.8 s	1.2 s
Interpretability	0.2	0.4	0.8	0.9

## Data Availability

The raw data supporting the conclusions of this article will be made available by the authors on request.

## Abbreviations

The following abbreviations are used in this manuscript:

ERr-CIC	Evidential reasoning rule considering causality-informed correlation
CCM	Convergent cross-mapping
CHCC	Conditionally hybrid correlation coefficient
PAMD	Photoelectric angle measurement device
